# Integrated DFT and Cyclic Voltammetry Approach for Screening Redox-Active Knoevenagel Adducts with Potential Antioxidant Applicability

**DOI:** 10.1021/acsomega.6c00610

**Published:** 2026-05-06

**Authors:** Pedro P. C. Santos, Ivanete C. Palheta, Lucas F. Araújo, Roberto M. Bezerra, Irlon M. Ferreira, David E. Q. Jimenez, Cleydson B. R. Santos, Francisco D. Silva, Ryan S. Ramos

**Affiliations:** † Laboratory of Modeling and Computational Chemistry, Department of Biological and Health Sciences, Federal University of Amapá, Macapá 68902-280, Amapá, Brazil; ‡ Laboratório de Química Analítica e Inorgânica, 681196 Universidade do Estado do Amapá, Macapá 68901-258, Amapá, Brazil; § Laboratório de Biocatálise e Síntese Orgânica Aplicada, 74364 Universidade Federal do Amapá, Macapá 68903-419, Amapá, Brazil; ∥ Núcleo de Estudos e Seleção de Moléculas Bioativas, Instituto de Ciências da Saúde, Universidade Federal do Pará, Belém 66075-110, Brazil; ⊥ Laboratory of Bioprospection and Atomic Absorption, Federal University of Amapá, Macapá 68903-419, Amapá, Brazil

## Abstract

In this study, a combined quantum-chemical and electrochemical approach was employed to evaluate synthesized Knoevenagel adducts as potential antioxidant candidates. Molecular geometries were optimized at the B3LYP/6-31 + G­(d,p) level, and electronic descriptors, including highest occupied molecular orbital, lowest unoccupied molecular orbital, energy gap, ionization potential (IP), single-electron transfer (SET), and molecular electrostatic potential (MEP) maps, were obtained from single-point calculations at the B3LYP/6-311++G­(2d,2p) level. Analysis of the MEP maps together with the frontier molecular orbitals enabled the identification of electron-rich and electron-deficient regions and helped rationalize the preferred redox-active sites involved in electron-transfer-based antioxidant screening. The theoretical results showed that dopamine, used as the reference compound, exhibited the lowest IP among the analyzed molecules. Among the Knoevenagel adducts, compound 6 showed the most favorable profile, with an IP of 182.92 kcal mol^–1^ and a SET value of 4.92 kcal mol^–1^. A series of Knoevenagel adducts (1–6) was synthesized by microwave-assisted condensation of cyanoacetic acid with aromatic aldehyde derivatives in the presence of KOH, affording yields of 71–85%. Structural elucidation was performed by Fourier transform infrared spectroscopy (FTIR) and nuclear magnetic resonance spectroscopy. For compounds 5 and 6, FTIR spectra confirmed the characteristic C≡N stretching bands at 2223 and 2221 cm^–1^, respectively, as well as C=O absorptions at 1687 and 1716 cm^–1^; compound 6 additionally showed bands consistent with methoxy substitution. Electrochemical validation was performed by cyclic voltammetry using graphite/epoxy composite electrodes chemically modified with compounds 5 and 6. The 6/GRAPHITE/EPOXY electrode exhibited the highest redox response, with anodic and cathodic peak currents of 59.25 and −40.37 μA, respectively, indicating more efficient electron transfer than the 5/GRAPHITE/EPOXY and unmodified electrodes. Overall, the results demonstrate that the integration of DFT-based screening with cyclic voltammetry is an effective strategy for identifying redox-active Knoevenagel derivatives with potential antioxidant applicability.

## Graphical abstract

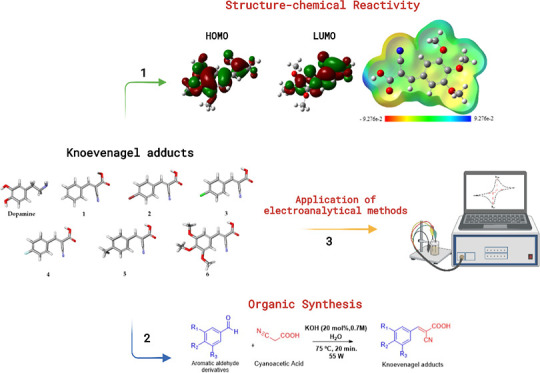

## Introduction

Free radicals, defined as chemical species containing one or more unpaired electrons, play a central role in several biological and pathological processes, being directly associated with oxidative stress.^1,2^ Among the main radicals of biological interest, species such as the hydroxyl radical (^•^OH), the superoxide anion (O_2_^•‑^) and the peroxyl radical (ROO^•^) stand out, which, in excess, can cause damage to lipids, proteins, and nucleic acids, triggering a series of disorders, including cancer, neurodegenerative, and cardiovascular diseases.^3,4^ Because antioxidant behavior is a multifactorial phenomenon, its evaluation cannot rely on a single experimental or theoretical parameter. In this context, density functional theory (DFT) has been widely used to estimate redox- and reactivity-related descriptors, such as ionization potential, electron affinity, frontier orbital energies, and molecular electrostatic potential, which are useful for rationalizing electron-transfer propensity and identifying potentially active sites.

In this context, oxidative stress, characterized by an imbalance between pro-oxidant species and antioxidant defense systems, has driven the search for compounds capable of scavenging free radicals and mitigating their deleterious effects.^5^ Compounds derived from the Knoevenagel reaction, generated through the condensation of aldehydes with active methylene compounds, have attracted increasing interest due to their conjugated systems and functional groups, which promote free-radical stabilization and may confer antioxidant and larvicidal activities.^6,7^ The Knoevenagel reaction is one of the most versatile methods for C–C bond formation and has been widely applied in the synthesis of biologically relevant molecules, including pharmaceuticals, natural products, and polymeric materials. In this sense, Knoevenagel adduct derivatives (Figure 1) are referred to as building blocks because they participate in the synthesis of molecules with antibacterial, antineoplastic, antimicrobial, and anti-Alzheimer’s activity.^7^

**Figure 1:**
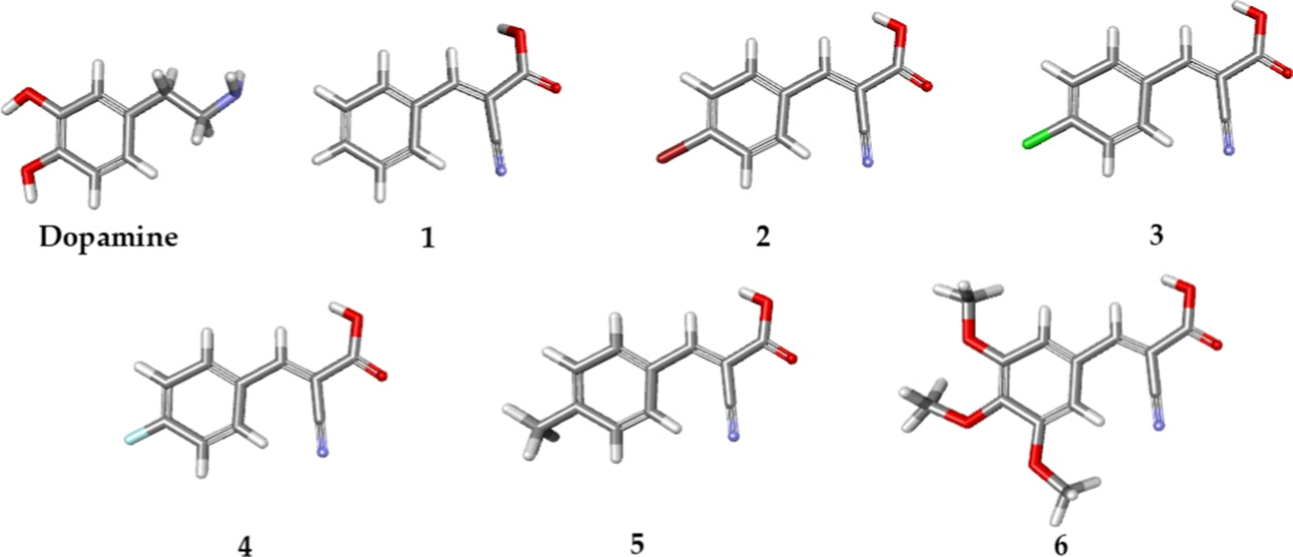
Optimized structure of dopamine and the Knoevenagel adducts (**1–6**) by the B3LYP/6-31 + G­(d,p) basis set. Carbon (gray); hydrogen (white); oxygen (red); nitrogen (blue); bromine (wine); chlorine (green); fluorine (cyan).

In addition, computational chemistry tools based on DFT enable the prediction of molecular parameters associated with antioxidant reactivity, including frontier molecular orbital energies [highest occupied molecular orbital (HOMO) and lowest unoccupied molecular orbital (LUMO)], IP, electron affinity, and spin-density distribution in radical species.^8^ Dopamine, owing to the presence of catechol groups (*ortho*-dihydroxybenzene), exhibits high antioxidant capacity, acting predominantly through single-electron transfer (SET), hydrogen atom transfer (HAT), and sequential proton loss electron transfer (SPLET) mechanisms, particularly under physiological pH conditions, where deprotonation of the phenolic groups facilitates electron transfer and stabilizes the resulting radicals by resonance within the aromatic ring.^8^ However, Knoevenagel adducts, which feature α,β-unsaturated systems conjugated with electron-withdrawing groups and may also contain phenolic substituents, can exert antioxidant activity not only through the classical SET, HAT, and SPLET mechanisms, but also through a distinct pathway based on Michael-type radical addition, whereby nucleophilic radicals add to the β-carbon of the conjugated system, yielding stable products and contributing to the mitigation of oxidative stress.^9,10^

The evaluation of the antioxidant activity of chemical compounds can be performed by several analytical methods, among which conventional colorimetric tests, such as DPPH (2,2-diphenyl-1-picrylhydrazyl), ABTS (2,2′-azinobis-3-ethylbenzothiazoline-6-sulfonic acid), FRAP (ferric reducing antioxidant power), and ORAC (oxygen radical absorbance capacity), are widely used due to their simplicity, low cost, and applicability in complex matrices.^11–13^ However, these methods present several limitations, including solvent dependence, susceptibility to interfering species, nonphysiological experimental conditions, and limited ability to distinguish among different antioxidant mechanisms. In this regard, cyclic voltammetry has emerged as a valuable complementary electroanalytical approach, providing redox parameters such as oxidation potentials, number of electrons transferred, and radical-product stability, while also enabling real-time evaluation of the kinetics and thermodynamics of these processes.^14–16^

This integrated theoretical–experimental approach provides a rational screening framework for correlating DFT-derived electron-transfer descriptors with cyclic voltammetry redox parameters, thereby supporting the identification of the most redox-active Knoevenagel adducts and their potential antioxidant applicability. In this work, the main originality lies in the use of graphite/epoxy composite electrodes directly modified with the synthesized Knoevenagel adducts, without relying on multicomponent architectures or nanomaterials commonly employed in electroanalytical platforms. In addition, although arylidene malononitrile and cyanoacetate derivatives related to the Knoevenagel reaction have previously been investigated in conventional antioxidant assays, the combined use of DFT-based descriptor screening and electrochemical validation by cyclic voltammetry for this class of compounds remains underexplored. Thus, the present study offers a useful methodological contribution to the mechanistically guided screening of redox-active Knoevenagel derivatives.^17,18^

## Results and Discussion

FMOs were analyzed to compare the spatial distribution of the electron-donating (HOMO) and electron-accepting (LUMO) regions across the series. The HOMO is mainly delocalized over the aromatic/conjugated π-framework and, in hydroxylated derivatives, also shows contribution from oxygen lone pairs (*n*O) coupled to the π-system, which is consistent with easier oxidation (electron donation). In contrast, the LUMO is predominantly localized on the electron-deficient segment of the α,β-unsaturated moiety (β-carbon and carbonyl/cyano region), indicating the preferred sites for electron uptake (π character).^19–21^ From this perspective, the orbitals shown in Figure 2 were obtained.

**Figure 2:**
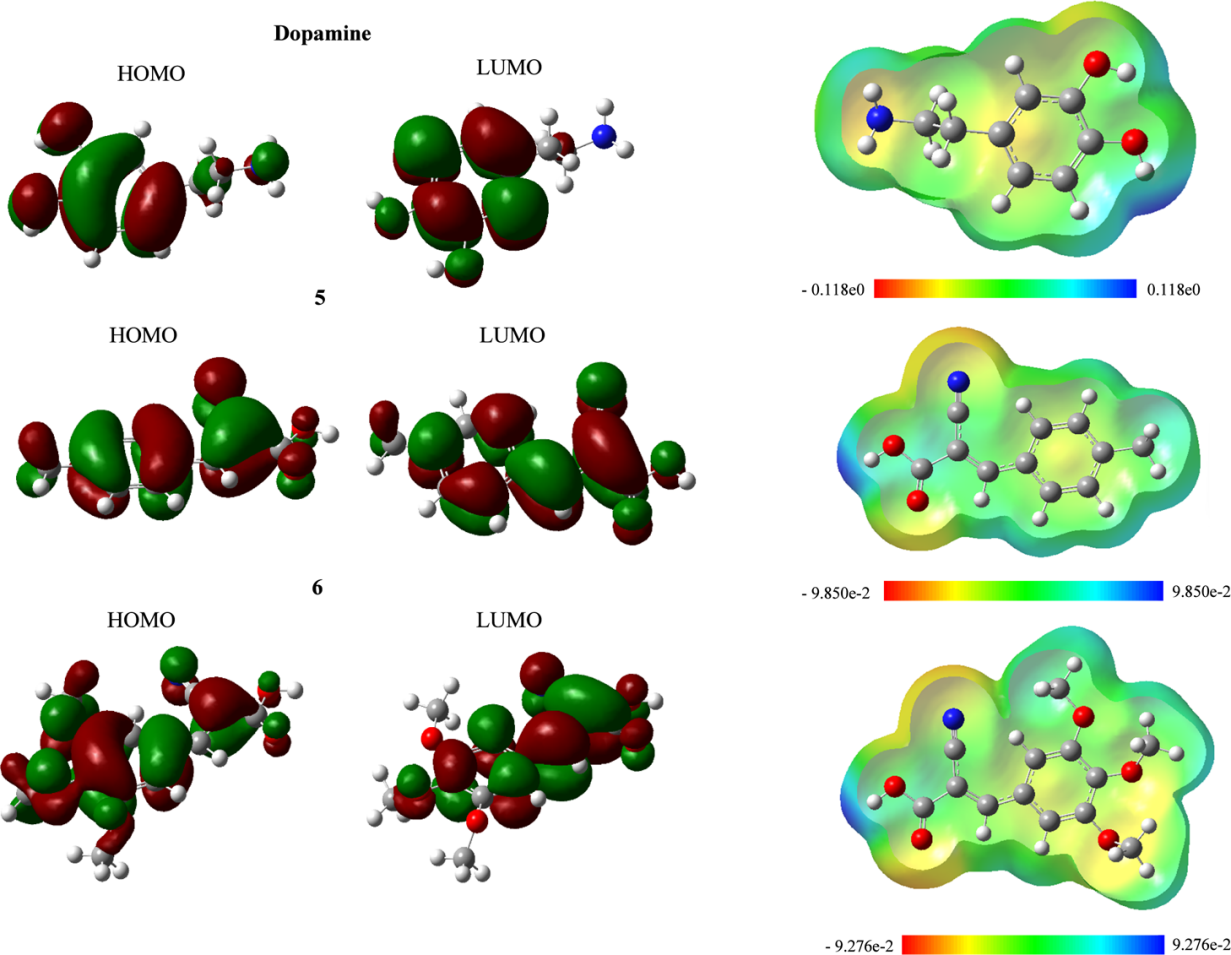
HOMO, LUMO, and MEP of dopamine and Knoevenagel adduct-derived molecules.

The data revealed nucleophilic regions within the aromatic ring of dopamine and the Knoevenagel derivatives. This feature is expected, since the π bonds between carbon atoms promote electron delocalization through resonance throughout the ring.^22,23^ The observed interactions are relatively weak, indicating low stability. Therefore, in the presence of another molecule, these bonds may be cleaved, generating carbon-centered anionic species rather than releasing hydrogen. In addition, the methyl groups display less pronounced electron-rich regions, suggesting that these moieties are electronically more neutral than other regions of the molecule. Furthermore, the results obtained allowed the calculation of the GAP, IP, and SET, as shown in Table 1.

**Table 1:** Quantum Chemical Properties of Molecules Using the B3LYP/6-311++G­(2d,2p) Level of Theory

molecules	HOMO (eV)	LUMO (eV)	GAP (eV)	IP (kcal/mol)	SET (kcal/mol)
dopamine	–8.90	–4.87	4.03	178.00	0.00
1	–7.23	–3.00	4.24	205.70	27.70
2	–7.17	–3.20	4.01	202.00	24.00
3	–7.21	–3.20	4.06	203.60	25.60
4	–7.41	–3.22	4.20	209.92	31.92
5	–6.99	–2.87	4.12	198.87	20.87
6	–6.41	–2.77	3.64	182.92	4.92

The control molecule used was dopamine, and the values of the parameters used were obtained based on the optimization of the DFT method, according to results obtained in the literature.^24^ Compared to the control molecule, which has a high antioxidant potential, the HOMO and LUMO values were lower for all Knoevenagel adduct derivatives. From the perspective of the energy difference (EQ), which determines the reactivity of a chemical entity, it was observed that compound **5** had a higher modulus while the control had an intermediate value.

The IP can be defined as the minimum energy for an electron to be torn from the atom to which it belongs.^25,26^ From this perspective, the lower the energy required to remove an electron, the greater the antioxidant potential of the molecule. Accordingly, compound 6 exhibited the lowest ionization energy among the Knoevenagel derivatives with a value of 182.92 kcal mol^–1^. This finding is relevant for this class of compounds, although the value remains slightly higher than that observed for dopamine (178.00 kcal mol^–1^).

SET is associated with the ability to retain free radicals, which is directly related to antioxidant activity when its values are as low as possible.^27,28^ Compound 6 exhibited the lowest value among the molecules analyzed. Despite this, dopamine showed greater free-radical scavenging potential, which may be attributed to its molecular structure, as it favors dehydrogenation in electrophilic regions. Furthermore, the electrostatic potential maps (EPMs) revealed hydroxyl groups with more positively charged hydrogen atoms, suggesting a higher propensity for homolytic bond cleavage.^29,30^ Therefore, owing to the presence of two hydroxyl groups (–OH), dopamine is more susceptible to oxidation than the compounds investigated in this study. Nevertheless, compound 6 showed promising results and may be further optimized through structural modifications or molecular simplification. Because antioxidant behavior in biological systems is multifactorial, the conclusions of this study are limited to the redox descriptors and electrochemical responses obtained under the experimental conditions employed.

The infrared spectrum of molecule 5 showed a band corresponding to the formation of axial bonds in the cyano group (C≡N) at 2223.6 cm^–1^ and the C=O interaction at 1687.8 cm^–1^. Furthermore, bands corresponding to vibrations in the C=C bonds of the aromatic ring were blocked at 1580.6 cm^–1^. The band at 739.8 cm^–1^ also revealed the bonds that the four hydrogens form in the same aromatic region. Classification of the obtained band was made according to results found in the literature (Table 2). Proton nuclear magnetic resonance of molecule 5 (500 MHz, DMSO-*d*_6_) demonstrated that at 8.27 ppm (s, 1H) of vinyl hydrogen, at 7.93 ppm (d, *J* = 8.0 Hz, 2H), which belong to the hydrogens of the aromatic ring, at 7.39 ppm (d, *J* = 8.0 Hz, 2H), which belong to the hydrogens of the aromatic ring and a singlet at 2.38 ppm (s, 3H) which corresponds to the hydrogens present in the methyl.

**Table 2:** Classification of Bands Found in Fourier Transform Infrared Spectroscopy (FTIR)

	molecule (λ = cm^–1^)		Refs 7 and 31	
interaction	5	6		
4H	739	-	-	-
CN	2223	2221	2229	2223
C=C (ARO)	1580	1576	1604	1581
C–O–H	1409	1421	1422	-
C=O	1687	1716	1728	1728
C–O–C	-	1250	-	-

The infrared spectrum of compound 6 exhibited a band attributed to the stretching vibration of the cyano group (C≡N) at 2221.5 cm^–1^, together with a band assigned to the C=O stretching vibration at 1716.6 cm^–1^. As also observed for compound 5, bands related to the C=C stretching vibrations of the aromatic ring were detected at 1576.5 cm^–1^. The band at 1421.9 cm^–1^ was associated with C–O–H vibrations, which, in combination with the carbonyl absorption at 1716.6 cm^–1^, suggests the presence of a carboxylic acid functionality. In addition, the band at 1250.9 cm^–1^ indicated the presence of methoxy groups interacting with the aromatic ring (Table 2).

Thus, proton nuclear magnetic resonance of molecule 6 (500 MHz, DMSO-*d*_6_) demonstrated the presence of a singlet at 8.28 ppm (s, 1H) of the vinyl hydrogen, a singlet at 7.49 ppm (s, 2H) characteristic of the hydrogens of the aromatic ring, a singlet at 3.87 ppm (s, 6H) of the hydrogens characteristic of the methoxyl groups, and at 3.78 ppm (s, 3H) of the hydrogens characteristic of the methoxyl groups. Dopamine (DP) oxidation–reduction voltammograms were obtained on carbon paste electrodes with and without modification by means of cyclic voltammetry (CV), with a DP concentration equal to 1 mM and a support electrolyte of HClO_4_ (perchloric acid) of 0.1 M. Thus, cyclic voltammetry experiments were carried out with electrodes modified with Knoevenagel adducts 5 and 6, together with the unmodified GRAPHITE/EPOXY electrode, to evaluate the effect of these compounds on electrode modification and, consequently, to investigate their redox behavior and potential antioxidant relevance, as shown in Figure 3.

**Figure 3:**
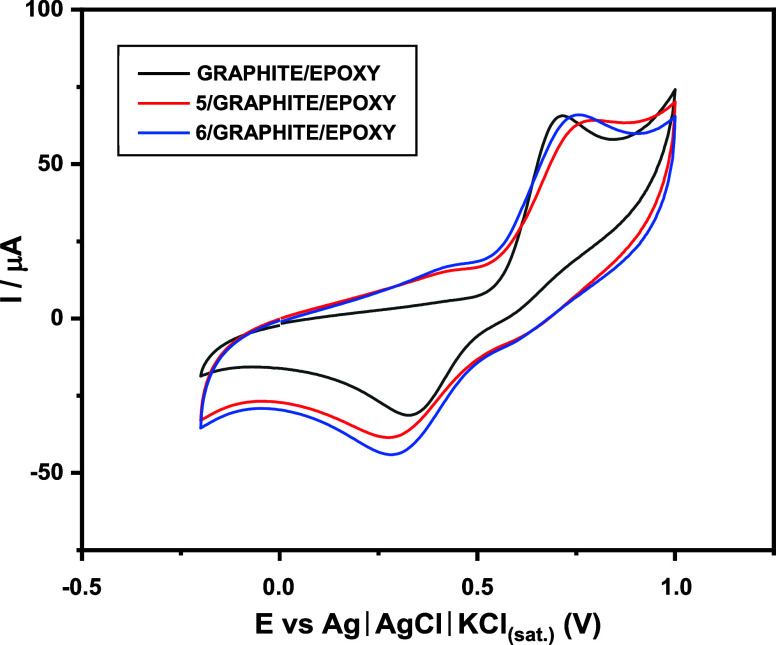
Cyclic voltammetry of dopamine in a modified electrode of (—) 5/GRAPHITE/EPOXY, (—) 6/GRAPHITE/EPOXY, and (—) unmodified.

The data showed that the 6/GRAPHITE/EPOXY electrode exhibited a cathodic peak current (*I*p) 2.87-fold higher than that of the unmodified electrode. Similarly, the 5/GRAPHITE/EPOXY electrode also displayed an enhanced cathodic peak current, 1.85-fold higher than that of the unmodified electrode; however, its response remained lower than that observed for the electrode modified with compound 6, in agreement with the in silico findings. Modification of the composites enhanced the electrocatalytic behavior of the system, and the higher cathodic peak currents were associated with increased electron-transfer rate constants (*K*s) compared to those of the electrode unmodified with Knoevenagel adducts.

With respect to peak separation, the unmodified GRAPHITE/EPOXY electrode exhibited a Δ*E*p of 0.39 V, corresponding to a smaller separation than those observed for composites 5 and 6, which presented values of 0.52 and 0.40 V, respectively. The values obtained for the anodic potential (*E*pa), cathodic potential (*E*pc), peak separation (Δ*E*p), and anodic (*I*pa) and cathodic (*I*pc) peak currents agreed with those reported by Freire (2015), as presented in Table 3.

**Table 3:** Data Values Obtained by CV of *I*p, *E*p, and Δ*E*_p_ for Dopamine and Compounds **5** and **6**

electrode	*E*_pa_ (V)	*E*_pc_ (V)	Δ*E*_p_ (V)	*I*_pa_(μA)	*I*_pc_(μA)	|*I*_pa_/*I*_pc_|
GRAPHITE/EPOXY	0.71	0.34	0.37	24.81	–14.05	1.77
5/GRAPHITE/EPOXY	0.79	0.27	0.52	40.23	–26.05	1.54
6/GRAPHITE/EPOXY	0.75	0.28	0.47	59.25	–40.37	1.47

The study interval to characterize the kinetics of the redox process was delimited between scanning speeds of 10 to 650 mV s^–1^, which demonstrated a linear variation in peak currents (*I*p) as the speed increased. Thus, the increase in *I*p supports the assumption regarding the reversibility of the redox process for the 5/GRAPHITE/EPOXY and 6/GRAPHITE/EPOXY electrodes, despite the peaks not exhibiting complete symmetry (Figure 4). Based on the electrochemical parameters, the anodic (*E*_pa_) and cathodic (*E*_pc_) peak potentials were determined, allowing for the calculation of the peak potential difference (Δ*E*p), which gradually increased with the scan rate.

**Figure 4:**
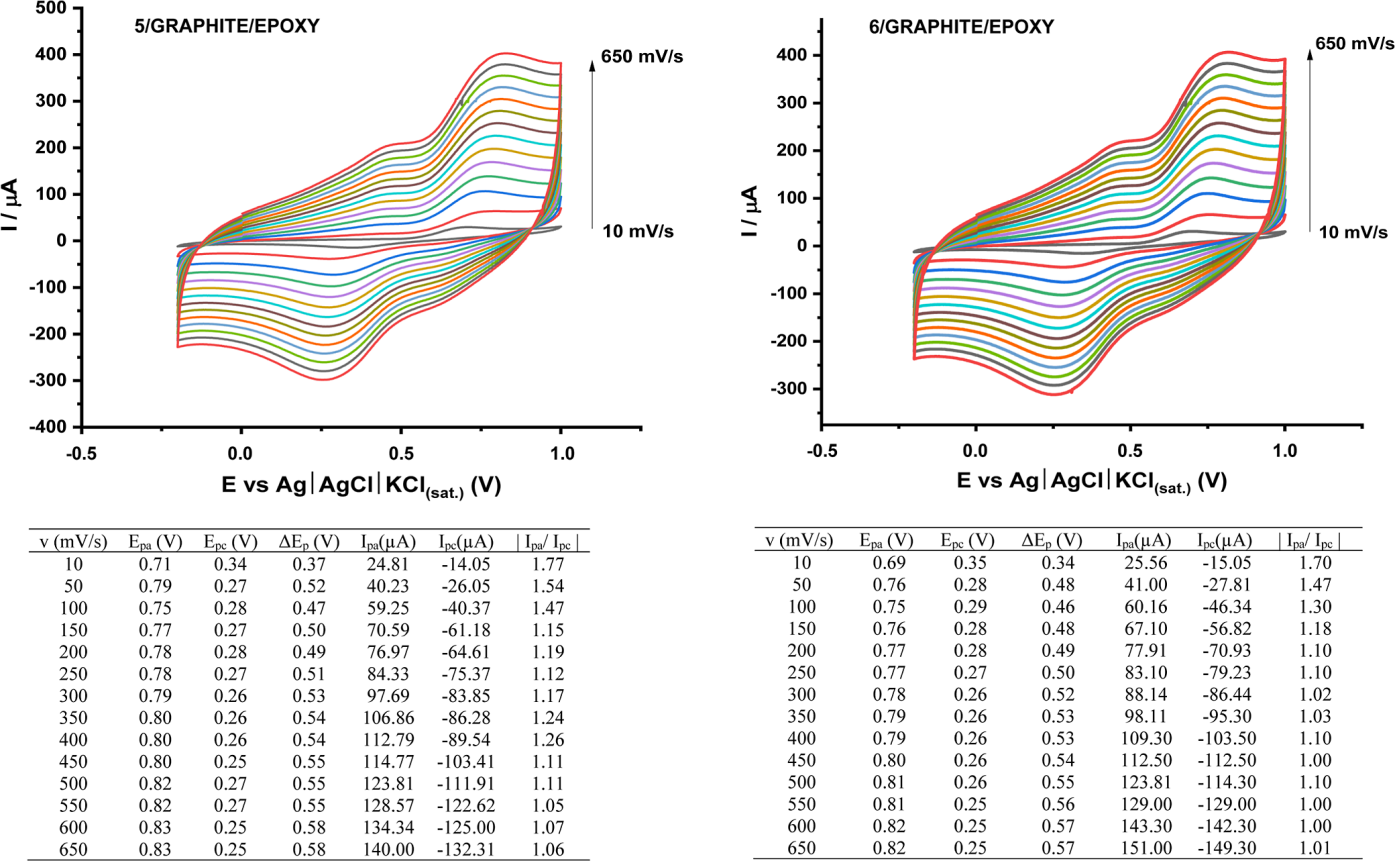
Variation of voltametric parameters as a function of the potential scan speed (*V*) and the electrochemical standard found.

The graphs of peak current by the square root of the sweep speed and peak current by the log of the speed were obtained, to understand the mass transport of the DP in the modified electrodes, as shown in Figure 5. Thus, it was found that the peak current increased in relation to *v* ^1/2^, which varied linearly for electrodes 5 (**A**) and 6 (**B**) (*R* ^2^ > 0.98), which indicates that the process is more reversible. The relationship between the logarithm of the peak current and the log of the velocity provides information about the process occurring at the electrode surface. Thus, modified electrodes 5 (**C**) and 6 (**D**) presented an angular coefficient (δ log (*i*_p_)/δ log­(*v*)) equal to 0.43 in the linear regression, which is a condition for a process controlled only by diffusion, because it is closer to 0.5.^32,33^ Therefore, the process is diffusional and does not present adsorption on the surface of the modified electrode.

**Figure 5:**
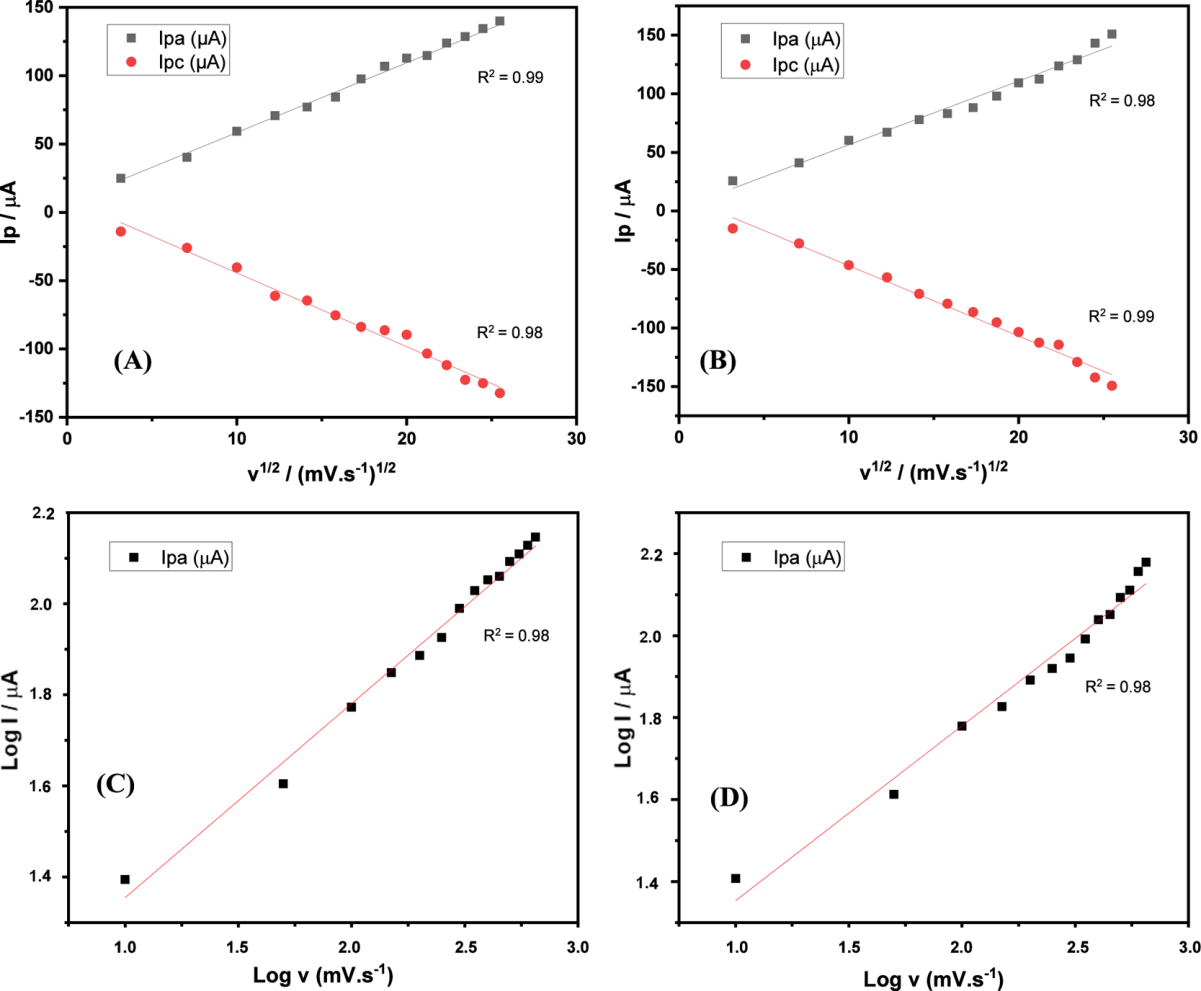
Anodic and cathodic peak currents as a function of scan rate. (**A–C**) Modified electrode-compound **5**; (**B–D**) modified electrode-compound **6**.

## Methods

### Structure-Chemical Reactivity of Knoevenagel Adducts

The Knoevenagel adducts are represented in Figure 1 and are obtained by the reaction between cyanoacetic acid and different aromatic aldehyde derivatives. Geometric optimization of all structures was performed using DFT calculations at the B3LYP/6-31 + G­(d,p) level. Analysis of the relationship between chemical structure and antioxidant activity (electronic properties) was conducted based on additional calculations at the B3LYP/6-311++G­(2d,2p) levels with double-ζ and triple-ζ bases.^34–36^ All theoretical calculations, including visualization of the MEP, were performed with the Gaussian 16 computational package.^37^ The chemical reactivity of the compounds was evaluated based on the energies of the frontier orbitals (HOMO and LUMO), which were associated with the nucleophilicity and electrophilicity parameters, respectively.^38^ The global reactivity parameter was estimated based on the energy difference between the LUMO and HOMO orbitals (gap^LUMO–HOMO^), as shown in eq 1, in addition to the spin density analysis.

1 $$GAP=E_{LUMO}-E_{HOMO}$$

The IP is related to electron or hydrogen donations. The IP was calculated as the energy difference between the neutral molecule (ArOH) and the respective cation free radical (ArOH^•+^), as shown in eq 2. Radical stability for electron or hydrogen transfers was determined by the related stabilization energy, as shown in eq 3, for single electron transfers (SETs) and mechanisms compared to dopamine (DOH).

2 $$IP=E_{{ArOH}^{\cdot +}}-E_{ArOH}$$

3 $$SET=\left[E_{{ArOH}^{\cdot +}}+E_{DOH}\right]-\left[E_{ArOH}+E_{{DOH}^{\cdot +}}\right]$$

### Chemistry: General

Benzaldehyde (>99%) (100–52–7), 4-bromobenzaldehyde (99%) (1122–91–4), 4-chlorobenzaldehyde (97%) (104–88–1), 4-fluorobenzaldehyde (98%) (459–57–4), 4-methylbenzaldehyde (99%) (104–87–0), 4-nitrobenzaldehyde (99%) (555–16–8), 4-hydroxybenzaldehyde (98%) (123–08–0), 3,4,5-trimethoxybenzaldehyde (98%) (86–81–7), 4-methoxybenzaldehyde (97%) (123–11–5), 3-fluorobenzaldehyde (97%) (456–48–4), 2-fluorobenzaldehyde (97%) (446–52–6), 2-thiophenecarboxaldehyde (98%) (98–03–3), furfural (99%) (98–01–1), and cyanoacetic acid (99%) (372–09–8) were purchased from Sigma-Aldrich (Brazil-Sigma-Aldrich, Brazil) and used without further purification. The KOH (99%) (1310–58–3) and HCl (37%) (7647–01–0) were purchased from Synth (Diadema, SP, Brazil). The deuterated solvents, acetone-d6 (99%) (666–52–4), and DMSO-*d*_6_ (99%) (2206–27–1) were purchased from Cambridge Isotope Laboratories (Scielab, Rio de Janeiro, Brazil). The solvents hexane (99%) (110–54–3), ethyl acetate (99%) (141–78–6), acetone (99%) (6764–1), methanol (99%) (67–56–1), and ethanol (99%) (64–17–5) were purchased (Aldrich, Synth, Merck, and Vertec, SP, Brazil) and were used without further purification.

### Synthesis of Molecules (1–6)

The molecules were synthesized by the Organic Chemistry and Biocatalysis Laboratory of the Chemistry Institute of São Carlos (IQSC/USP), along with chemical elucidation information using spectroscopic techniques, such as ^1^H, ^13^C NMR, and FTIR. Aldehyde (1 mmol), cyanoacetic acid (1 mmol), and water (5 mL) were introduced into a microwave reactor (55 W). A KOH (0.7 M) solution (5.0 μL) was then added, and the reaction mixture was stirred at 75 °C for 20 min. The progress of the reaction was monitored by thin layer chromatography (TLC) using EtOAc/hexane (8:2) as the mobile phase. Upon completion, 1 mL of HCl (3 M) was added, and the mixture was further stirred for 30 min. The resulting mixture was extracted with ethyl acetate (3 × 25 mL), and the combined organic layers were concentrated under reduced pressure to dryness. The crude product was purified by column chromatography on silica gel (230–400 mesh) using the same eluent system as that employed for TLC (Figure 6) (Supporting Information-Figures S1–S6 and Tables S1 and S2). The molecular structures were used as input files (mol and sdf) for quantum chemical studies to optimize geometry and investigate molecular properties of antioxidant capacity. The molecules with the best antioxidant potentials, defined in the in silico step (IP e SET), were selected for experimental testing of redox potentials using the cyclic voltammetry (CV) technique.

**Figure 6:**
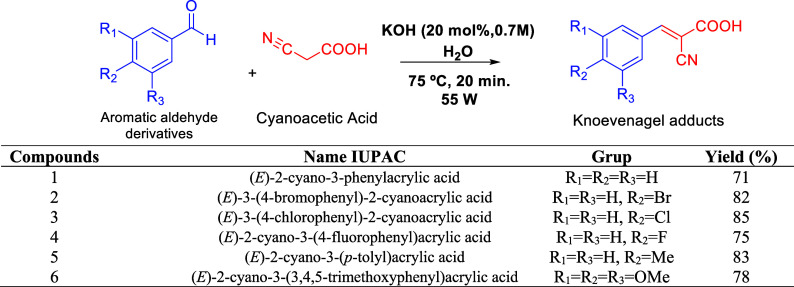
Syntactic scope of Knoevenagel adducts (**1–6**).

### Chemical Characterization by FTIR

Fourier transform infrared (FTIR) spectra were recorded using a Shimadzu IRAffinity-1 spectrometer model (Shimadzu, São Carlos, São Paulo, Brazil). Analyses were performed using compressed tablet disks prepared with KBr. The transmittance was expressed in cm^–1^ of the band between 4000 and 450 cm^–1^.

### ^1^H and ^13^C NMR Chemical Characterization

Nuclear magnetic resonance (NMR) spectra were recorded on an Agilent Technologies 500/54 Premium Shielded or Agilent Technologies 400/54 Premium Shielded spectrometer, with DMSO-*d*_6_ or acetone-*d*_6_ as solvent and tetramethylsilane (TMS) as the internal standard (Agilent Technologies, São Carlos, São Paulo, Brazil). The chemical shifts were expressed in ppm, and coupling constants (*J*) values were reported in Hz. The chemical shifts (δ) were expressed in parts per million (ppm) and referenced to the tetramethylsilane internal standard (TMS) signal and the deuterated solvent used DMSO-*d*_6_ (δ_H_: 2.50, δ_C_ 39.52). For ^1^H NMR spectra, the number of scans was 120, and for ^13^C NMR, it was was 881.

### Preparation of the Graphite/Epoxy Composite Electrode

Carbon paste electrodes are composite electrodes, consisting of graphite (conductor) and a resin (binder) in specific specifications.^39^ Graphite/epoxy composite electrodes were produced using a ratio of 35% epoxy resin and 65% graphite. (<20 μm, Sigma-Aldrich, Steinheim, Alemanha). Thus, 0.6789 g of EPOXY resin SQ-2126 (Avipol, São Paulo, Brazil) and 15 μL of EPOXY hardener SQ-3024 (Avipol, São Paulo, Brazil) were mixed with 1.2608 g of graphite, which formed a carbon paste.^40^ The paste was then placed in three 1 mL plastic syringes, and a copper wire was added to the tip of the plunger to establish electrical contact between the composite and the potentiostat. The composite was subjected to pressure using the plunger in a manual press, which remained in place for a day to cure the electrode. Finally, the electrodes were polished with 60- and 120-grit sandpaper for activation.

### Electroanalytical Reagents and Solutions

The reagents used were of analytical purity grade, which allowed the preparation of the electrochemical cell solution. The electrolytic solution was composed of perchloric acid [HClO_4_] (Êxodo Científica) as a supporting electrolyte at a concentration of 0.1 M and dopamine (DP) [C_8_H_11_NO_2_] (Sigma-Aldrich), at 1 mmol L^–1^, used as an electrochemical probe. The solutions used to modify the working electrode were alcoholic [C_2_H_6_O] (Dinâmica) (anhydrous alcohol), since molecules **5** and **6** do not solubilize in water, as Log *P* >1 (partition coefficients).

### Application of Electroanalytical Methods

#### Cyclic Voltammetry

Cyclic voltammetry measurements were performed at the Atomic Absorption and Bioprospection Laboratory—LAAB, of the Federal University of Amapá (UNIFAP), using an electrochemical cell connected to a Metrohm Autolab potentiostat/galvanostat model PGSTAT302N, controlled by Nova software, version 2.1.5, coupled to a computer suitable for establishing, compiling, and processing data and parameters (Table 4).^41^

**Table 4:** Parameters Used for Cyclic Voltammetry in Staircase

start potential (*V*_REF_)	0
upper vertex potential (*V*_REF_)	1.00
lower vertex potential (*V*_REF_)	–0.2
stop potential (*V*_REF_)	0
number of scans	3
scan rate (V s^–1^)	0.05
step (V)	0.00244

The electrochemical cell consisted of a proposed working electrode, chemically modified (GRAPHITE/EPOXY composite electrode) with two 5 μL layers for the test molecules, a miniaturized Ag|AgCl|KCl_(sat)_ reference electrode, and a platinum electrode as an auxiliary electrode, respectively.^42^ The data obtained was then processed using OriginPro software, version 9.65.^43^

## Conclusions

The quantum-chemical calculations used for structural optimization and energy minimization at the B3LYP/6-311++G­(2d,2p) level proved satisfactory, as they allowed the evaluation of the minimum energy required to remove an electron from compounds 5 and 6. The cyclic voltammograms confirmed the redox behavior of these systems, in agreement with the computed electron–loss parameters. In terms of electron transfer, the electrodes modified with the compounds exhibited higher cathodic peak current values than the unmodified graphite/epoxy electrode, indicating enhanced electron transfer from the electrode surface to the solution under identical scan-rate conditions. In addition, the computational results suggested that charge transfer occurs mainly through the hydroxyl groups, since the MEP maps revealed regions favorable to nucleophilic attack at these sites, as also observed for dopamine at its two hydroxyl groups, consistent with its greater antioxidant potential. Thus, the reliability of the methodology employed is further supported, with the aim of reducing reagent consumption and improving the efficiency of screening for new potential antioxidant agents. Nevertheless, the present findings should be interpreted as a computational–electrochemical screening rather than definitive biological evidence, since no cell-based or in vivo assays were conducted. In addition, solubility and medium-compatibility limitations of the Knoevenagel adducts may affect the electroanalytical response and restrict biological translatability. Therefore, future studies should incorporate orthogonal antioxidant assays (DPPH, ABTS, and FRAP), formulation strategies to enhance solubility, and biological validation in relevant experimental models.

## Supplementary Material



## Notes

All data supporting the results of this study, ^1^H NMR (500 MHz, DMSO-*d*_6_) of (*E*)-2-cyano-3-phenylacrylic acid (1); ^13^C NMR (125 MHz, DMSO-*d*_6_) of (*E*)-2-cyano-3-phenylacrylic acid (1); FT-IR of (*E*)-2-cyano-3-phenylacrylic acid (1); ^1^H NMR (500 MHz, DMSO-*d*_6_) of (*E*)-3-(4-bromophenyl)-2-cyanoacrylic acid (2); ^13^C NMR (125 MHz, DMSO-*d*_6_) of (*E*)-3-(4-bromophenyl)-2-cyanoacrylic acid (2); FT-IR of (*E*)-3-(4-bromophenyl)-2-cyanoacrylic acid (2); ^1^H NMR (400 MHz, DMSO-*d*_6_) of (*E*)-3-(4-chlorophenyl)-2-cyanoacrylic acid (3); ^13^C NMR (100 MHz, DMSO-*d*_6_) of (*E*)-3-(4-chlorophenyl)-2-cyanoacrylic acid (3); FT-IR of (*E*)-3-(4-chlorophenyl)-2-cyanoacrylic acid (3); ^1^H NMR (400 MHz, DMSO-*d*_6_) of (*E*)-2-cyano-3-(4-fluorophenyl)­acrylic acid (4); ^13^C NMR (100 MHz, DMSO-*d*_6_) of (*E*)-2-cyano-3-(4-fluorophenyl)­acrylic acid (4); FT-IR of (*E*)-2-cyano-3-(4-fluorophenyl)­acrylic acid (4); ^1^H NMR (500 MHz, DMSO-*d*_6_) of (*E*)-2-cyano-3-(p-tolyl)­acrylic acid (5); ^13^C NMR (125 MHz, DMSO-*d*_6_) of (*E*)-2-cyano-3-(p-tolyl)­acrylic acid (5); FT-IR of (*E*)-2-cyano-3-(p-tolyl)­acrylic acid (5); ^1^H NMR (500 MHz, DMSO-*d*_6_) of (*E*)-2-cyano-3-(3,4,5-trimethoxyphenyl)­acrylic acid (6); ^13^C NMR (125 MHz, DMSO-*d*_6_) of (*E*)-2-cyano-3-(3,4,5-trimethoxyphenyl)­acrylic acid (6); FT-IR of (*E*)-2-cyano-3-(3,4,5-trimethoxyphenyl)­acrylic acid (6); selected bond lengths (Å) and angles (°) for 1 and 6; and hydrogen bonds parameters for 1 and 6 (PDF)
