# Supplementary material for: Integrated DFT and Cyclic Voltammetry Approach for Screening Redox-Active Knoevenagel Adducts with Potential Antioxidant Applicability
Source: ACS Omega. 2026 May 6;11(19):28496–504. doi: 10.1021/acsomega.6c00610 (PMC13191557; doi:10.1021/acsomega.6c00610)
Supplement: Supplementary file 1 [file ao6c00610_si_001.pdf]

Integrated DFT and cyclic voltammetry approach for screening redox-active Knoevenagel Adducts with potential antioxidant Applicability

Pedro P. C. Santos[1,2,3]; Ivanete C. Palheta[4]; Lucas F. Araújo[1]; Roberto M. Bezerra[5], Irlon M. Ferreira[3]; David E. Q. Jimenez[3]; Cleydson B. R. Santos[1]*; Francisco D. Silva[2]; Ryan S. Ramos[1,2]

[1] Laboratory of Modeling and Computational Chemistry, Department of Biological and Health Sciences, Federal University of Amapá, 68902-280 Macapá, AP, Brazil; pedrocncc.eng@gmail.com (P.P.C.S) ryanquimico@gmail.com (R.S.R); lucas.fauro@unifap.br (L.F.A);

[2] Laboratório de Química Analítica e Inorgânica, Universidade do Estado do Amapá, 68901-258, Macapá, AP, Brazil; francisco.silva@ueap.edu.br (F.D.S)

[3] Laboratório de Biocatálise e Síntese Orgânica Aplicada, Universidade Federal do Amapá, 68903-419 Macapá-AP, Brazil; derteriom@unifap.br (D.E.Q.J)

[4] Núcleo de Estudos e Seleção de Moléculas Bioativas, Instituto de Ciências da Saúde, Universidade Federal do Pará, Belém 66075-110, Brazil; ivapalheta@gmail.com (I.C.P)

[5] Laboratory of Bioprospection and Atomic Absorption, Federal University of Amapá, Macapá 68903-419, AP, Brazil; messias@unifap.br (R.M.B)

* Correspondence: breno@unifap.br (C.B.R.S)

Supporting Information

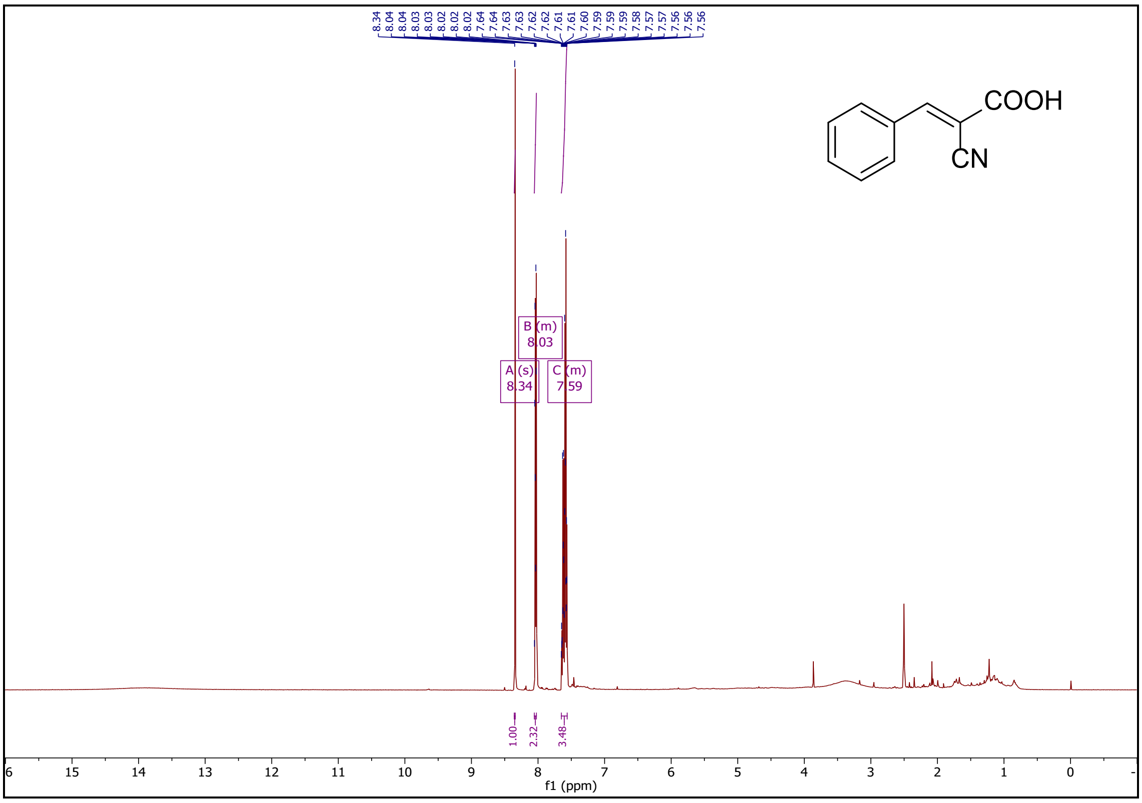

Figure S1. ^{1}H NMR (500 MHz, DMSO-d_6) of (*E)-2-cyano-3-phenylacrylic acid* (**1**).

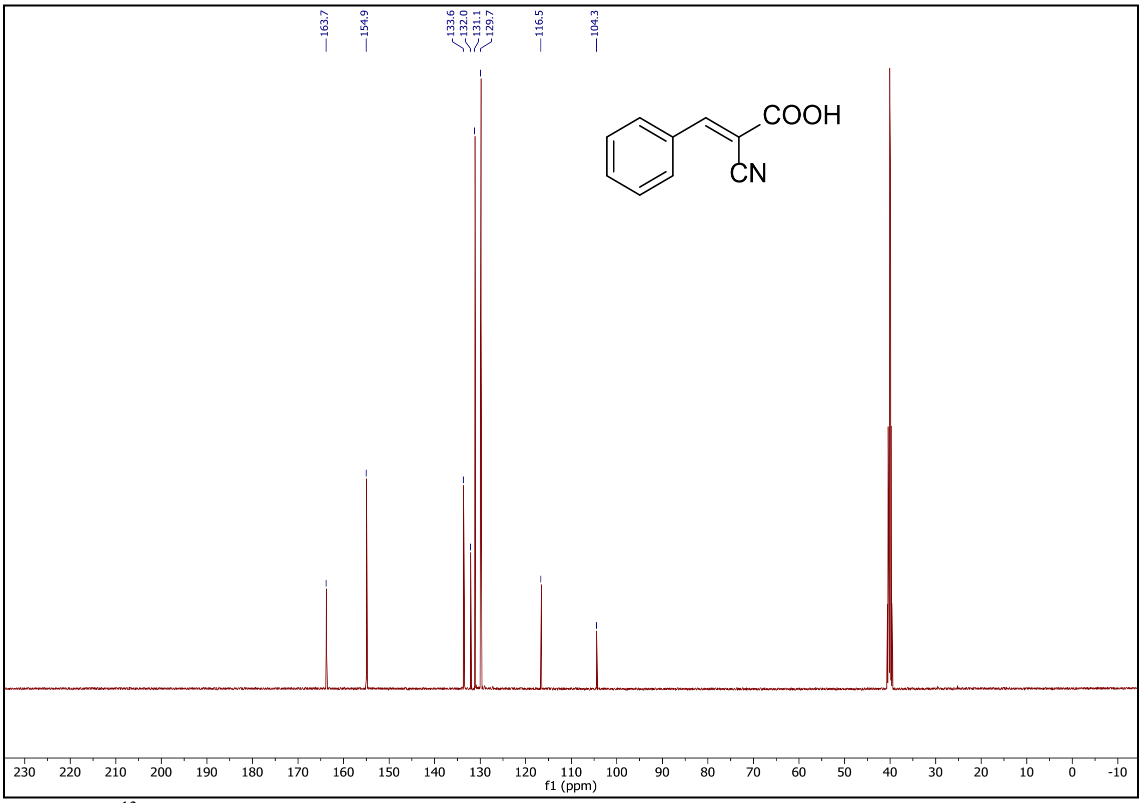

Figure S2.^{13}C NMR (125 MHz, DMSO-d_6) of (*E)-2-cyano-3-phenylacrylic acid* (**1**).

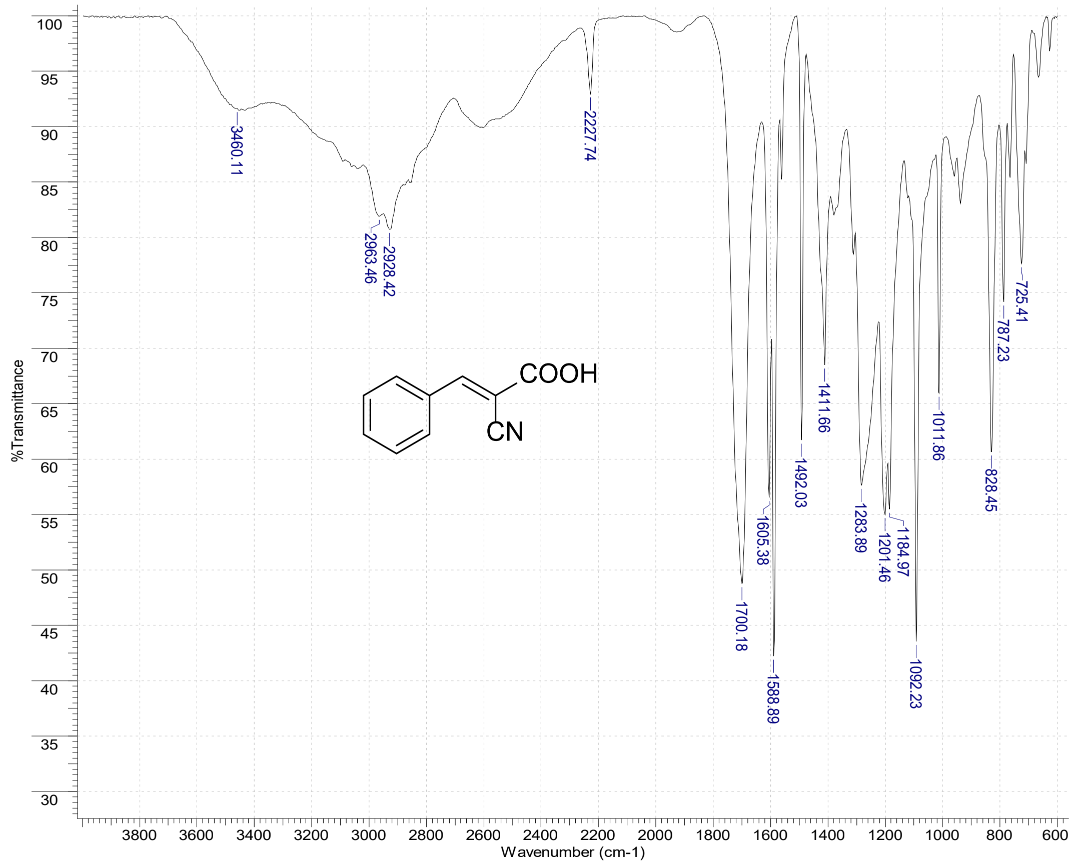

Figure S3. FT-IR of (*E*)-*2-cyano-3-phenylacrylic acid* (**1**).

(*E*)-2-cyano-3-phenylacrylic acid (**1**)

$C_{10}H_7NO_2$, 173.05 g.mol^{-1}; (71% yield); White crystal, m.p. = 179-182 ºC; FTIR ν_{max}(cm^{-1}) = 3460.11, 2963.46, 2928.42, 2227.74, 1700.18, 1605.38, 1588.89, 1492.03, 1411.66, 1283.89, 1201.46, 1184.97, 1092.23, 1011.86, 828.45, 787.23, 725.41.; ^{1}H NMR (500 MHz, DMSO-d_6) δ (ppm) = 8.34 (s, 1H), 8.04-8.01 (m, 2H), 7.64-7.56 (m, 3H).; ^{13}C NMR (125 MHz, DMSO-d_6) δ (ppm) = 163.7, 154.9, 133.6, 132.0, 131.1, 131.1, 129.7, 116.5, 104.3.

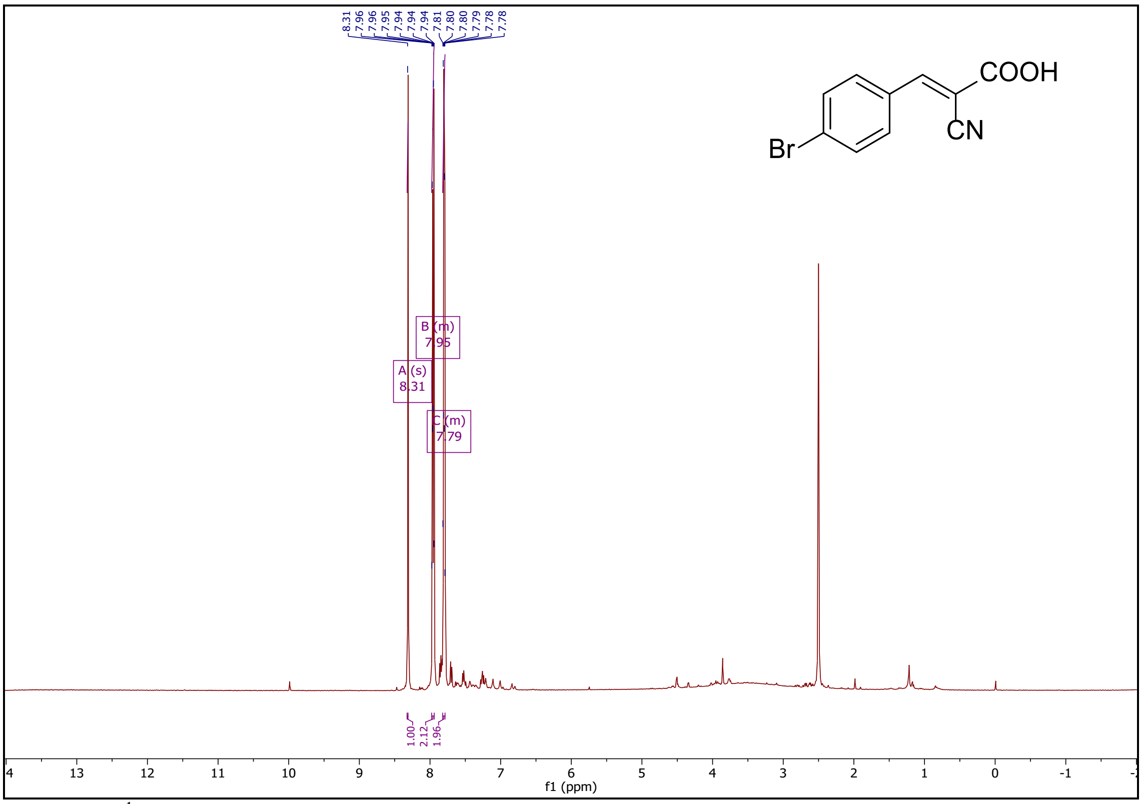

Figure S4. ^{1}H NMR (500 MHz, DMSO-d_6) of *(E)-3-(4-bromophenyl)-2-cyanoacrylic acid* (**2**).

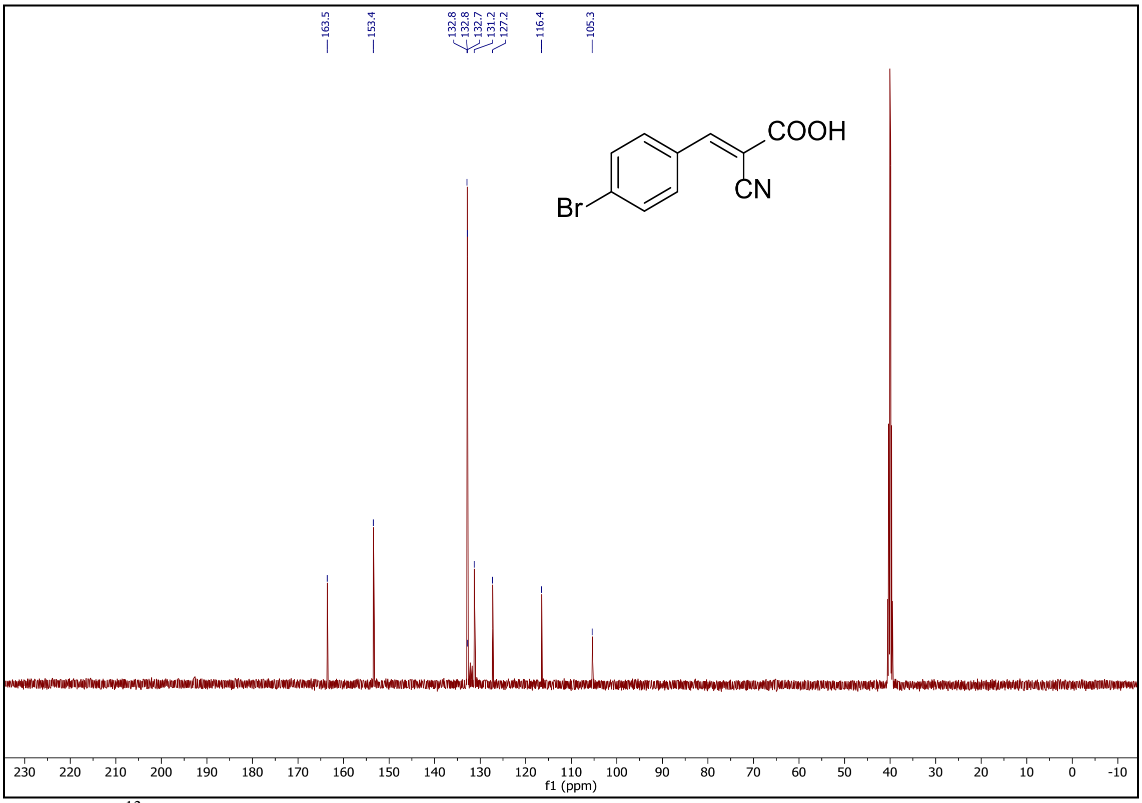

Figure S5. ^{13}C NMR (125 MHz, DMSO-d_6) of *(E)-3-(4-bromophenyl)-2-cyanoacrylic acid* (**2**).

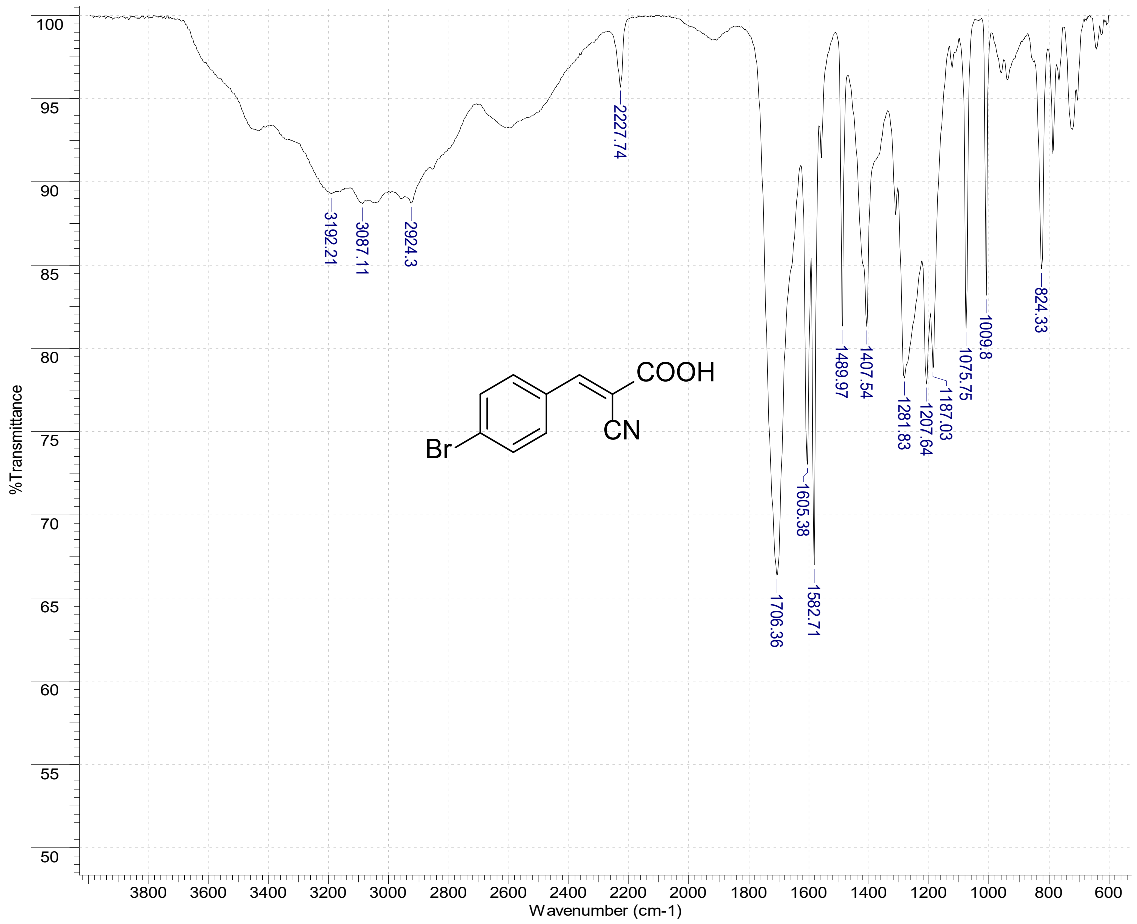

Figure S6. FT-IR of *(E)-3-(4-bromophenyl)-2-cyanoacrylic acid* (**2**).

(*E*)-3-(4-bromophenyl)-2-cyanoacrylic acid (**2**)

$C_{10}H_6NBrO_2$, 250.96 g.mol^{-1}; (82% yield); Yellow solid, m.p. = 179-182 ºC; FTIR ν_{max}(cm^{-1}) = 3192.21, 3087.11, 2924.3, 2227.74, 1706.36, 1605.38, 1528.71, 1489.97, 1407.54, 1281.83, 1207.64, 1187.03, 1075.75, 1009.8, 824.33.; ^{1}H NMR (500 MHz, DMSO-d_6) δ (ppm) = 8.31 (s, 1H), 7.96-7.94 (d, J = 8.0 Hz, 2H), 7.81-7.78 (d, J = 8.0 Hz, 2H).; ^{13}C NMR (125 MHz, DMSO-d_6) δ (ppm) = 163.5, 153.4, 132.8, 132.7, 131.3, 127.2, 116.4, 105.3.

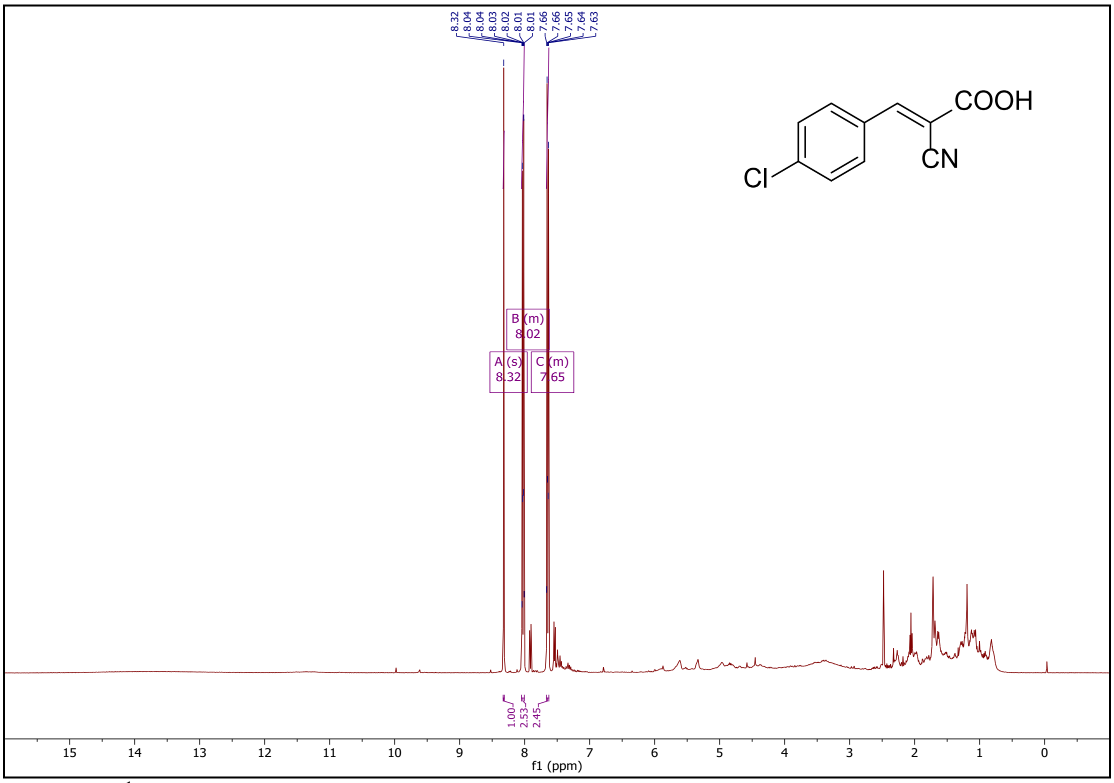

Figure S7. ^{1}H NMR (400 MHz, DMSO-d_6) of *(E)-3-(4-chlorophenyl)-2-cyanoacrylic acid* (**3**).

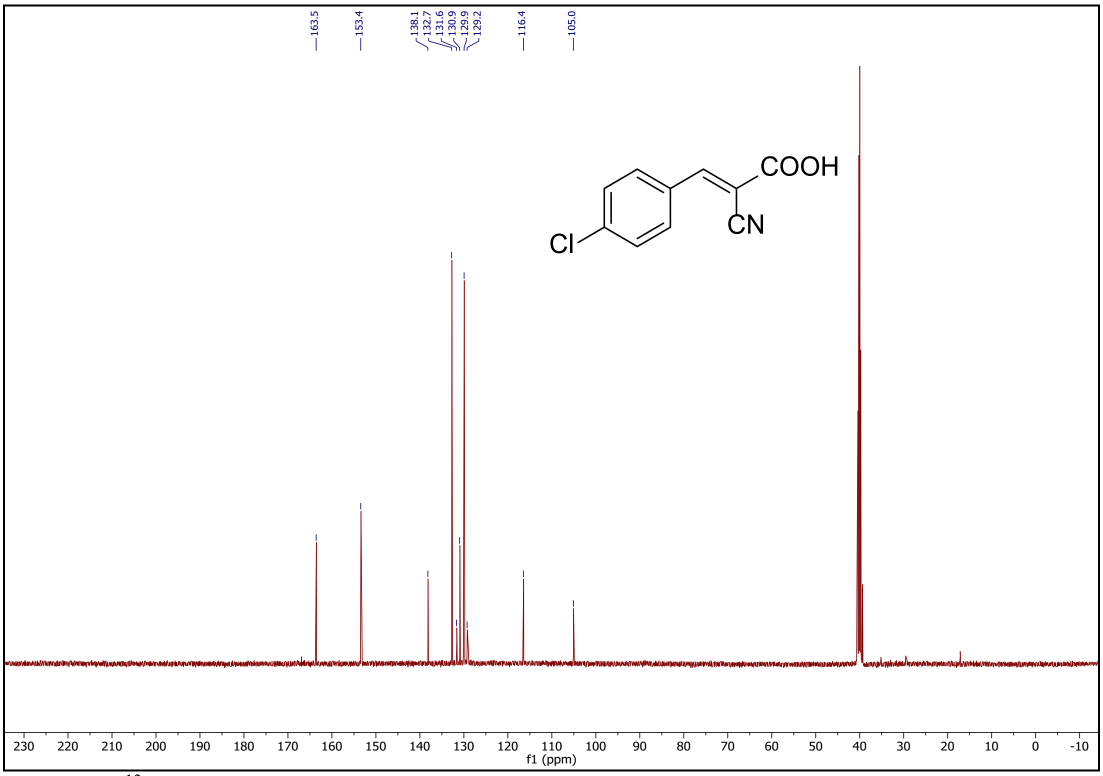

Figure S8. ^{13}C NMR (100 MHz, DMSO-d_6) of *(E)-3-(4-chlorophenyl)-2-cyanoacrylic acid* (**3**).

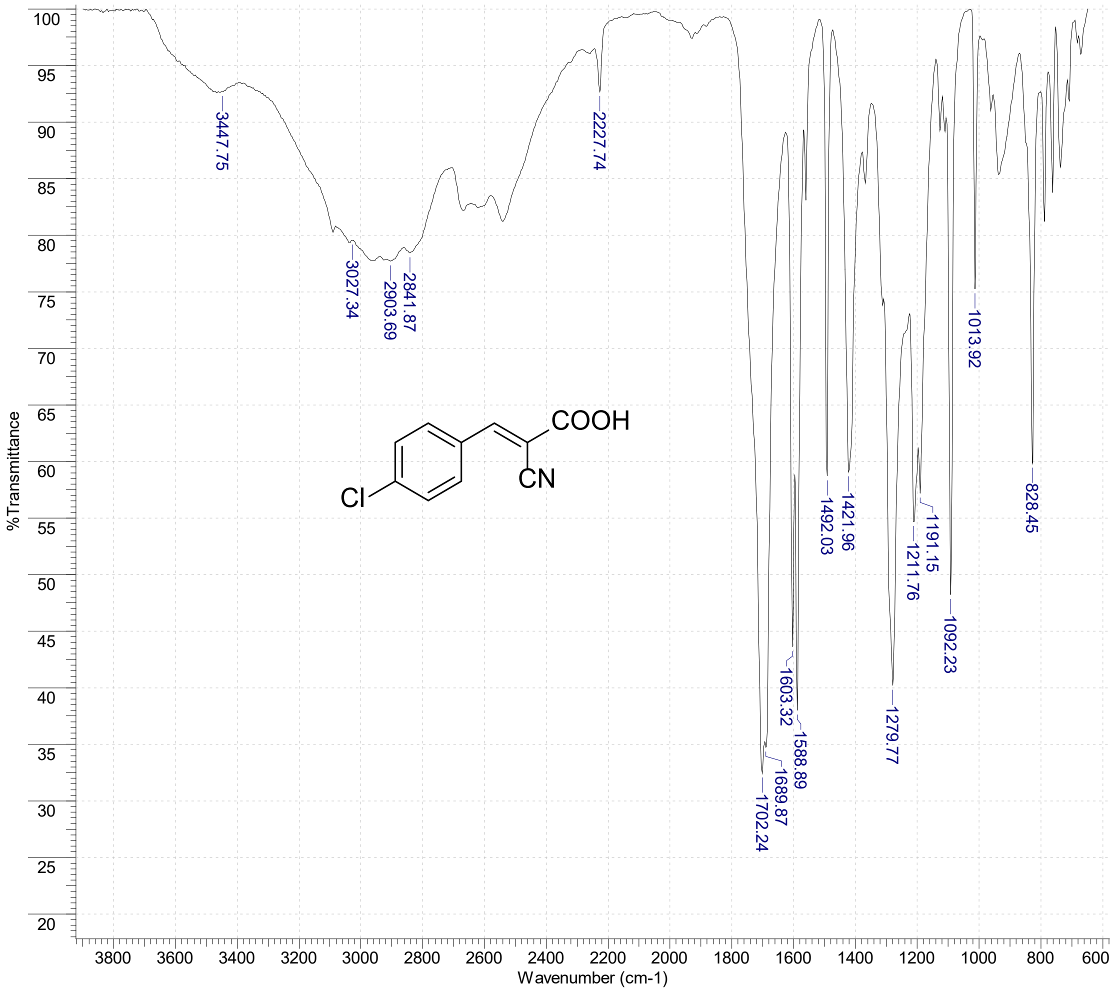

Figure S9. FT-IR of *(E)-3-(4-chlorophenyl)-2-cyanoacrylic acid* (**3**).

(*E*)-3-(4-chlorophenyl)-2-cyanoacrylic acid (**3**)

$C_{10}H_6NClO_2$, 207.01 g.mol^{-1}; (85% yield:); Yellow solid, m.p. = 192-194 ºC; FTIR ν_{max}(cm^{-1}) = 3447.75, 3027.34, 2903.69, 2841.87, 2227.74, 1702.24, 1689.87, 1492.03, 1588.89, 1492.03, 1421.96, 1279.77, 1211.76, 1191.15, 1092.23, 1013.92, 828.45.; ^{1}H NMR (400 MHz, DMSO-d_6) δ (ppm) = 8.32 (s, 1H), 8.04-8.00 (d, J = 8.0 Hz, 2H), 7.66-7.63 (d, J = 8.0 Hz, 2H).; ^{13}C NMR (100 MHz, DMSO-d_6) δ (ppm) = 163.5, 153.4, 138.1, 132.7, 131.6, 130.9, 129.9, 129.2, 116.4, 105.0.

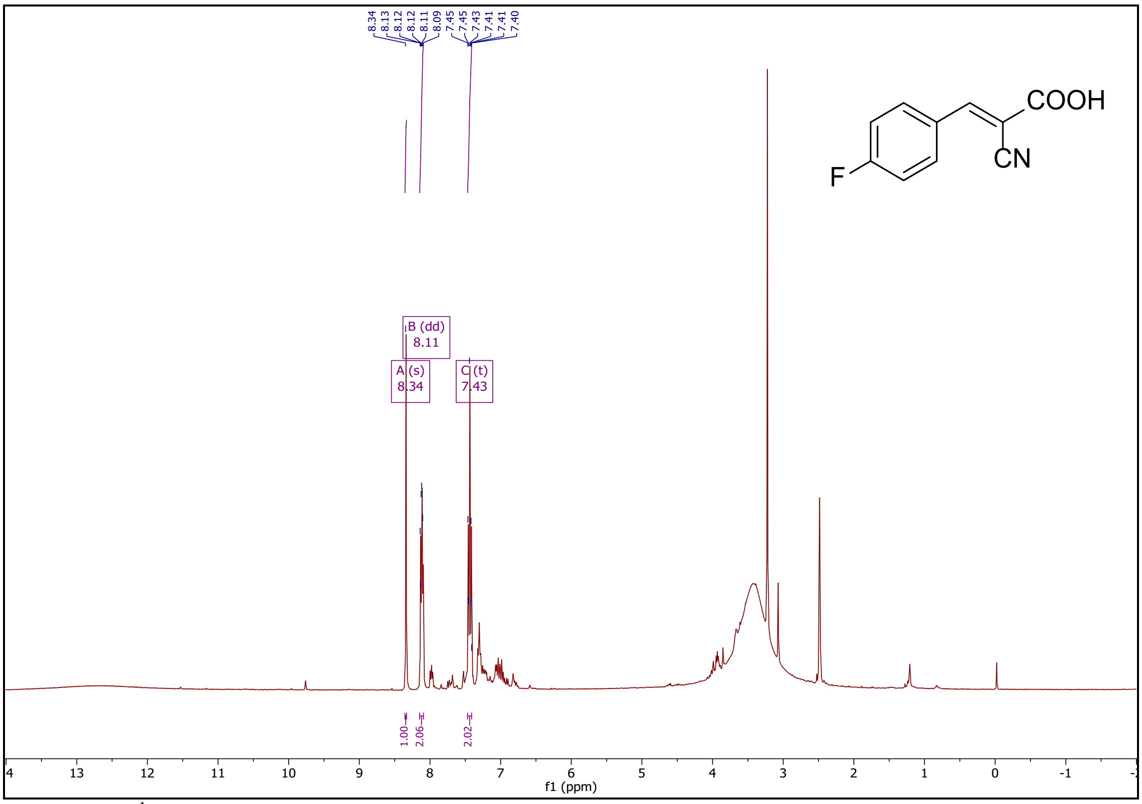

Figure S10. ^{1}H NMR (400 MHz, DMSO-d_6) of *(E)-2-cyano-3-(4-fluorophenyl)acrylic acid* (**4**).

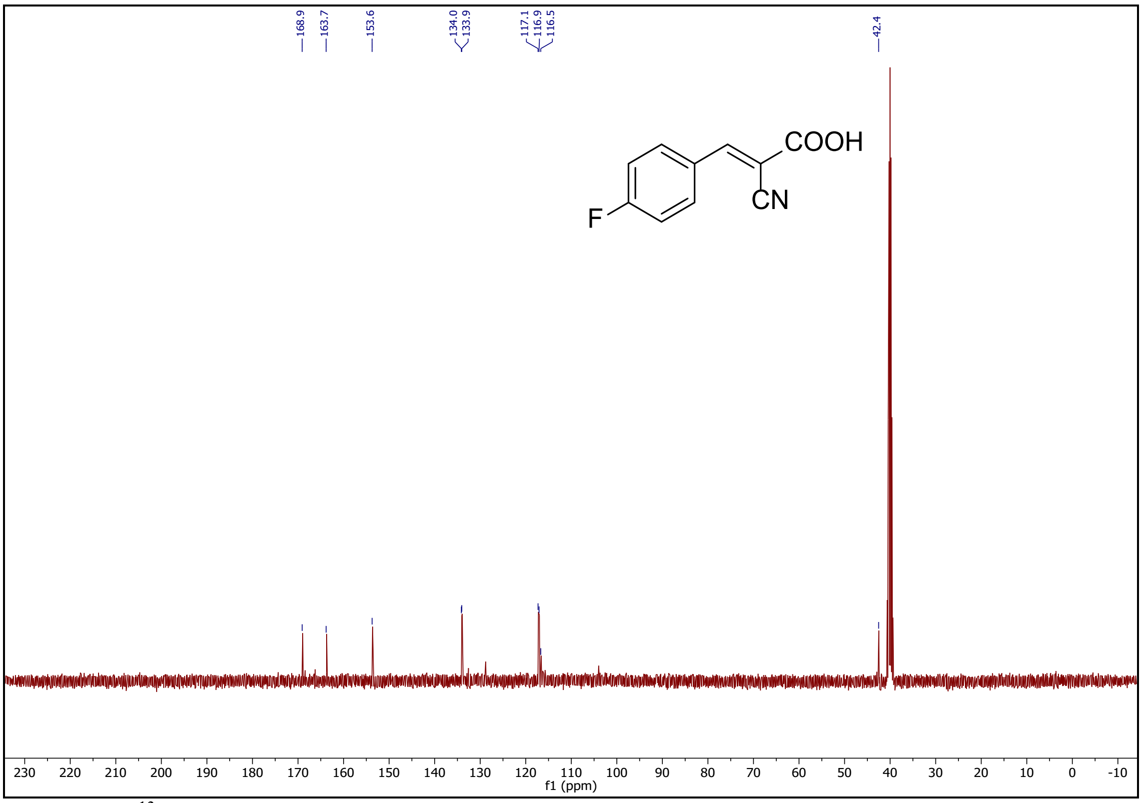

Figure S11. ^{13}C NMR (100 MHz, DMSO-d_6) of *(E)-2-cyano-3-(4-fluorophenyl)acrylic acid* (**4**).

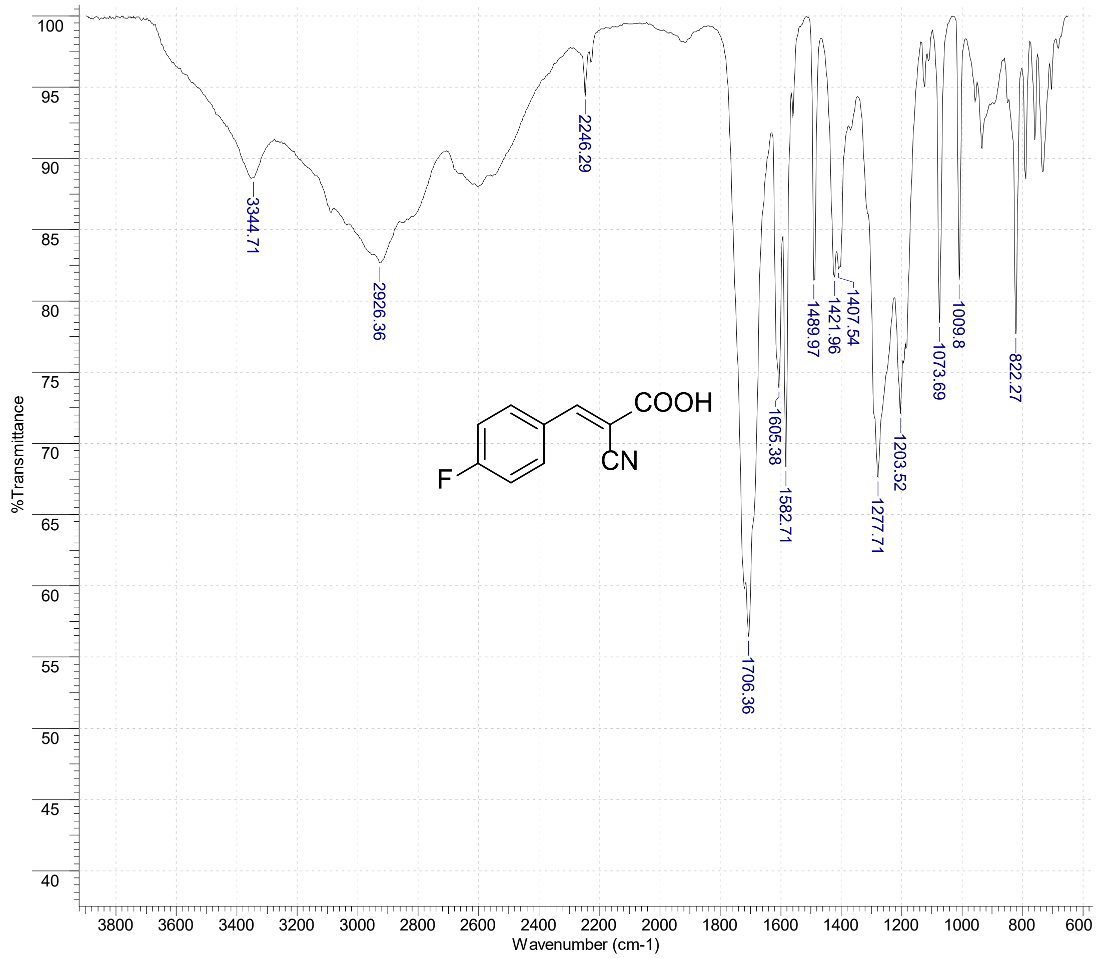

Figure S12. FT-IR of *(E)-2-cyano-3-(4-fluorophenyl)acrylic acid* (**4**).

(*E*)-2-cyano-3-(4-fluorophenyl)acrylic acid (**4**)

$C_{10}H_6NFO_2$, 191.04 g.mol^{-1}; (75% yield); Yellow solid, m.p. = 187-189 ºC; FTIR ν_{max}(cm^{-1}) = 3344.71, 2926.36, 2246.29, 1706.36, 1605.38, 1582.71, 1489.07, 1421.96, 1407.54, 1277.71, 1203.52, 1073.69, 1009.8, 822.27.; ^{1}H NMR (400 MHz, DMSO-d_6) δ (ppm) = 8.34 (s, 1H), 8.13-8.09 (dd, J = 8.7, 5.6 Hz, 2H), 7.45-7.41 (t, J = 8.8 Hz, 2H).; ^{13}C NMR (125 MHz, DMSO-d_6) δ (ppm) = 168.9, 163.7, 153.6, 134.0, 133.9, 128.7, 117.1, 116.9, 116.5, 103.9.

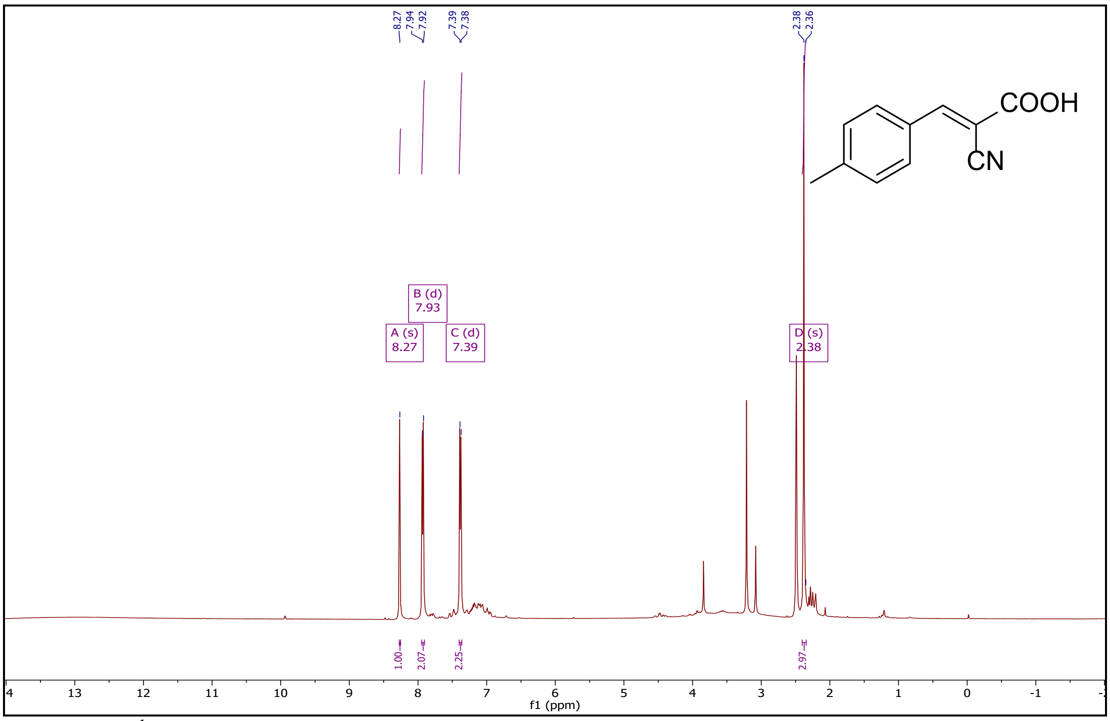

Figure S13. ^{1}H NMR (500 MHz, DMSO-d_6) of *(E)-2-cyano-3-(p-tolyl)acrylic acid* (**5**).

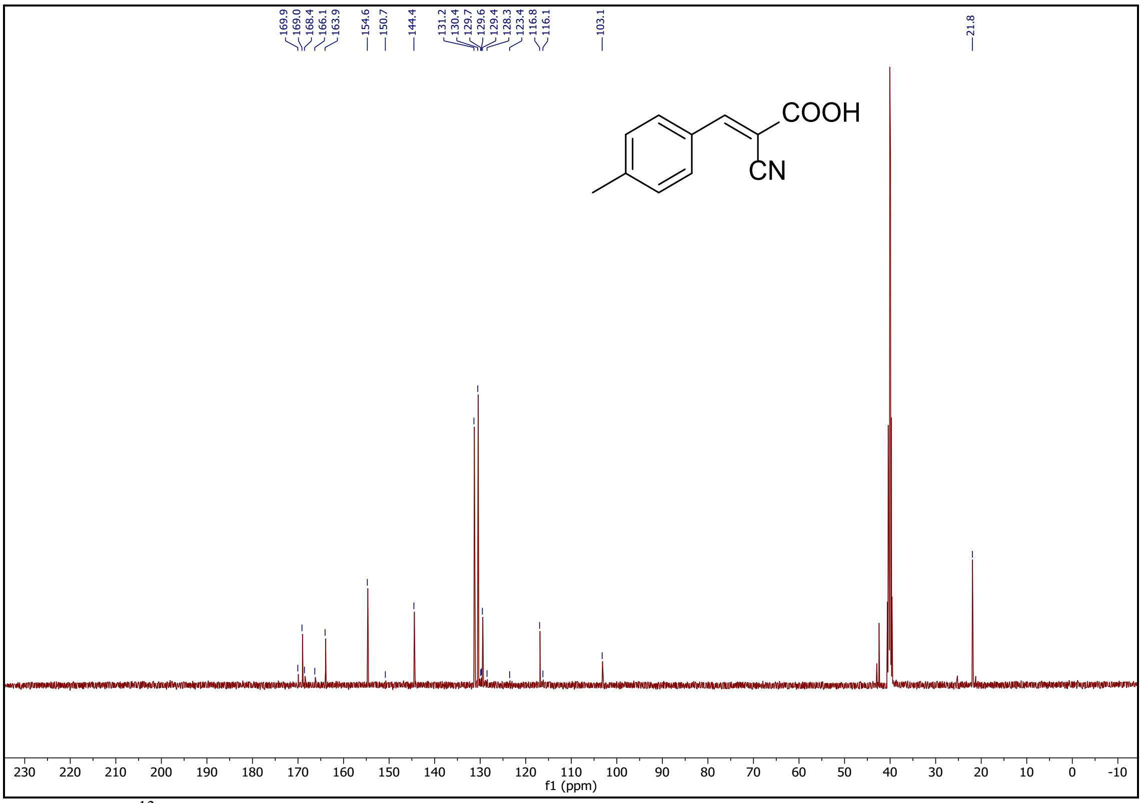

Figure S14. ^{13}C NMR (125 MHz, DMSO-d_6) of *(E)-2-cyano-3-(p-tolyl)acrylic acid* (**5**).

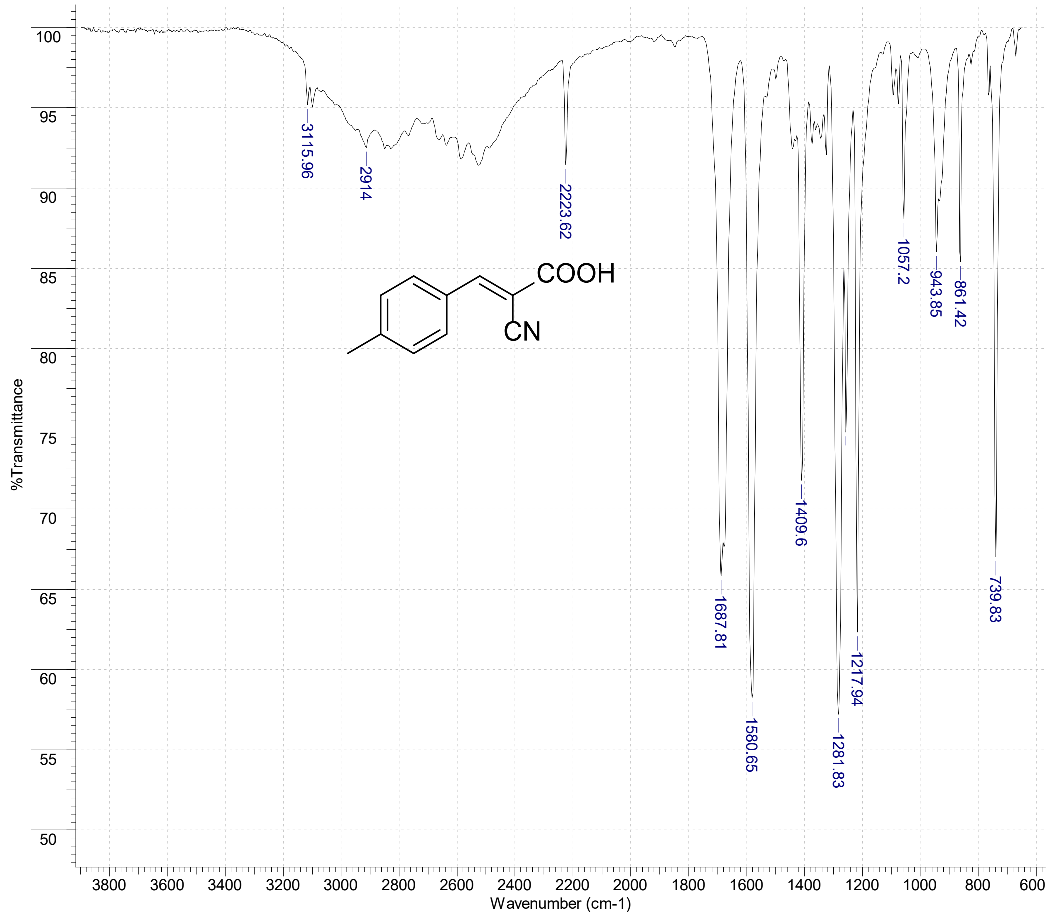

Figure S15. FT-IR of *(E)-2-cyano-3-(p-tolyl)acrylic acid* (**5**).

(*E*)-2-cyano-3-(*p*-tolyl)acrylic acid (**5**)

$C_{11}H_9NO_2$, 187.06 g.mol^{-1}; (83% yield); Yellow solid, m.p. = 187-189 °C; FTIR ν_{max}(cm^{-1}) = 3115.96, 2914, 2223.62, 1687.81, 1580.65, 1409.6. 1281.83, 1217.94, 1057.2, 943.85, 861.42. 739.83.; ^{1}H NMR (500 MHz, DMSO-d_6) δ (ppm) = 8.27 (s, 1H), 7.94-7.93 (d, J = 8.0 Hz, 2H), 7.39-7.38 (d, J = 8.0 Hz, 2H), 2.38 (s, 3H).; ^{13}C NMR (125 MHz, DMSO-d_6) δ (ppm) = 169.0, 164.0, 154.6, 144.4, 131.2, 130.4, 129.3, 116.8, 21,8.

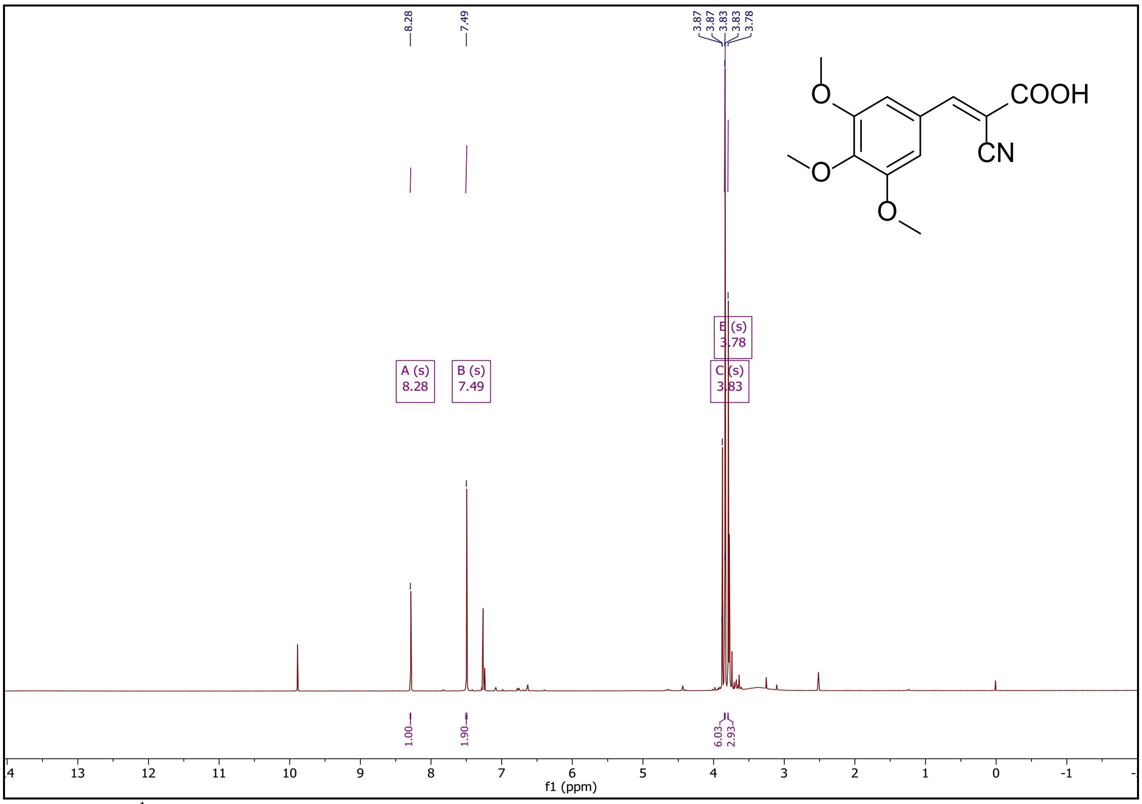

Figure S16. ^{1}H NMR (500 MHz, DMSO-d_6) of *(E)-2-cyano-3-(3,4,5-trimethoxyphenyl)acrylic acid* (**6**).

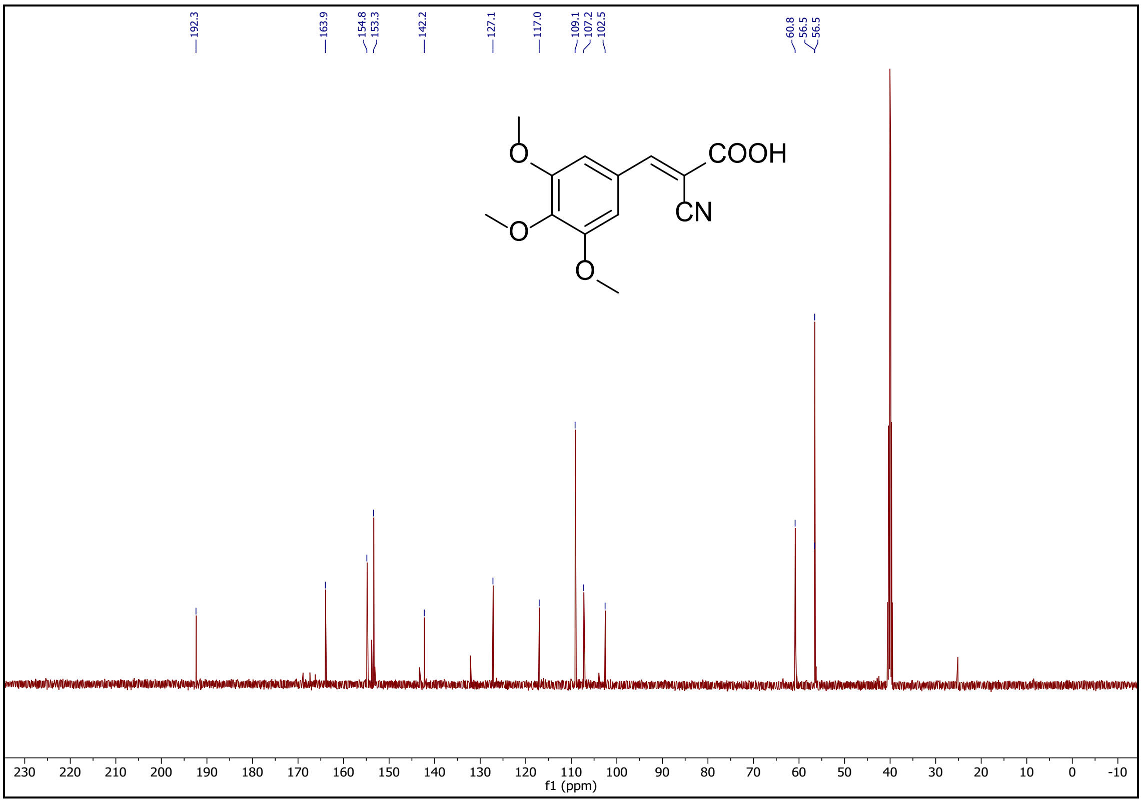

Figure S17. ^{13}C NMR (125 MHz, DMSO-d_6) of *(E)-2-cyano-3-(3,4,5-trimethoxyphenyl)acrylic acid* (**6**).

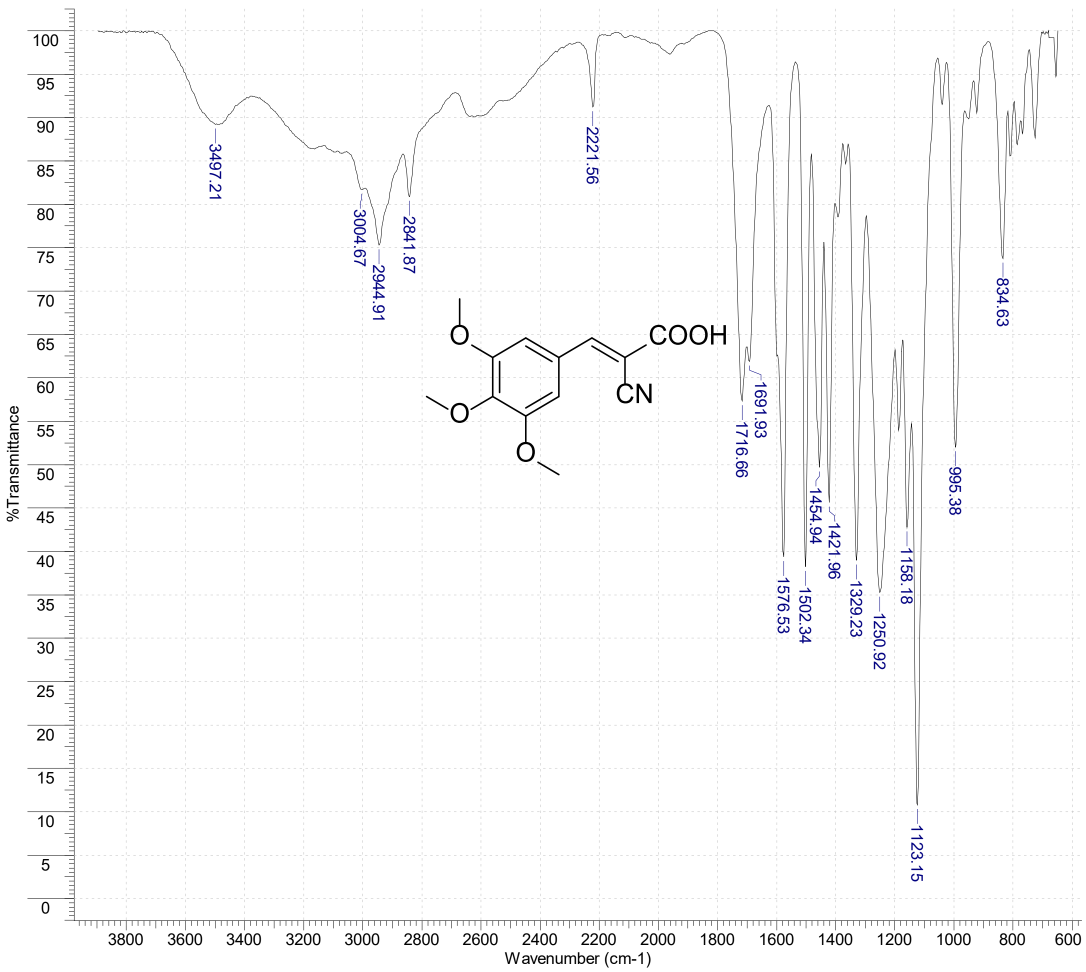

Figure S18. FT-IR of *(E)-2-cyano-3-(3,4,5-trimethoxyphenyl)acrylic acid* (**6**).

(*E*)-2-cyano-3-(3,4,5-trimethoxyphenyl)acrylic acid (**6**)

$C_{13}H_{13}NO_5$, 263.08 g.mol^{-1}; (78% yield); Yellow solid, m.p. = 206-208 ºC; FTIR ν_{max}(cm^{-1}) = 3497.21, 3004.67, 2944.91, 2841.87, 2221.56, 1716.53, 1691.93, 1576.53, 1502.34, 1454.04, 1421.96, 1329.23, 1250.92, 1158.18, 1123.15, 895.38, 834.63.; ^{1}H NMR (500 MHz, DMSO-d_6) δ (ppm) = 8.28 (s, 1H), 7.49 (s, 2H). 3.83 (s, 6H), 3.78 (s, 3H).; NMR ^{13}C (125 MHz, DMSO-d_6) δ (ppm) = 163.9, 154.8, 153.3, 142.2, 127.1, 116.9, 109.1, 107.2, 102.5, 60.8, 56.5, 56.5.

Table S1. Selected bond lengths (Å) and angles (º) for **1** and **6**.

	**1**	**6**
C1–O1	1.246(2)	1.243(5)
C1–O2	1.268(2)	1.254(5)
C2–C4	1.337(2)	1.348(5)
C3–N1	1.140(2)	1.131(6)
O1–C1–O2	124.4(1)	122.3(4)
C2–C4–C5	131.3(2)	131.6(4)
C2–C3–N1	178.1(2)	178.1(6)

Table S2. Hydrogen bonds parameters for **1** and **6**.

Compound	D–H···A	d(D–H)/ Å	d(H–A)/ Å	d(D–A)/ Å	D–H–A/º
1	O2–H2···O1^{i}	0.82	1.79	2.607(2)	173
	C7–H7···O1^{ii}	0.93	2.51	3.359(2)	151
6	O2–H2···O1^{iii}	0.82	1.79	2.597(5)	167
	C12–H12a···O5^{iv}	0.96	2.59	3.541(6)	169

Symmetry code: (i) 2–x, 1–y, 2–z; (ii) x, y, –1+z; (iii) –x, 1–y, 2–z; (iv) 2–x, –y, 1–z; (v) 1–x, –y, 1–z; (vi) -x, 1/2+y, 3/2–z; (vii) 1–x, 1–y, 1–z

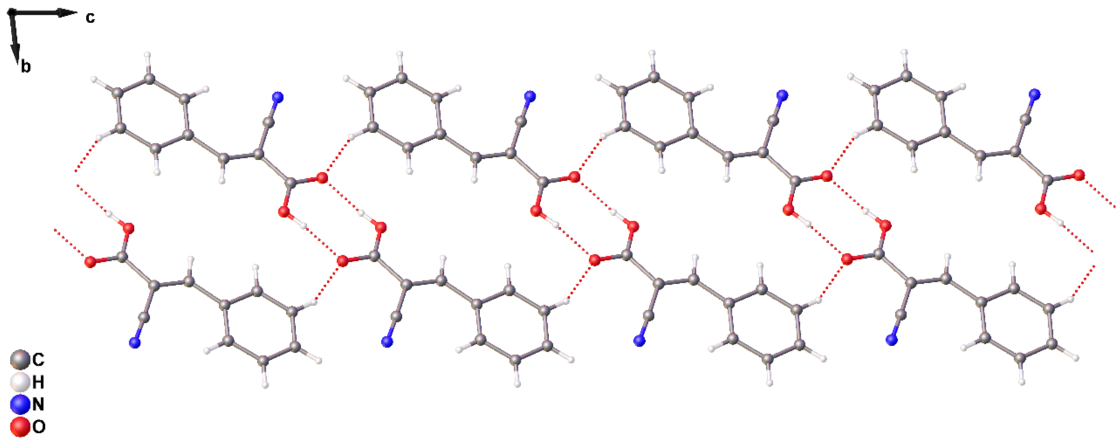

Figure S19. One-dimensional chain formed in **1** with the hydrogen bonds, viewed in the *bc* plane.

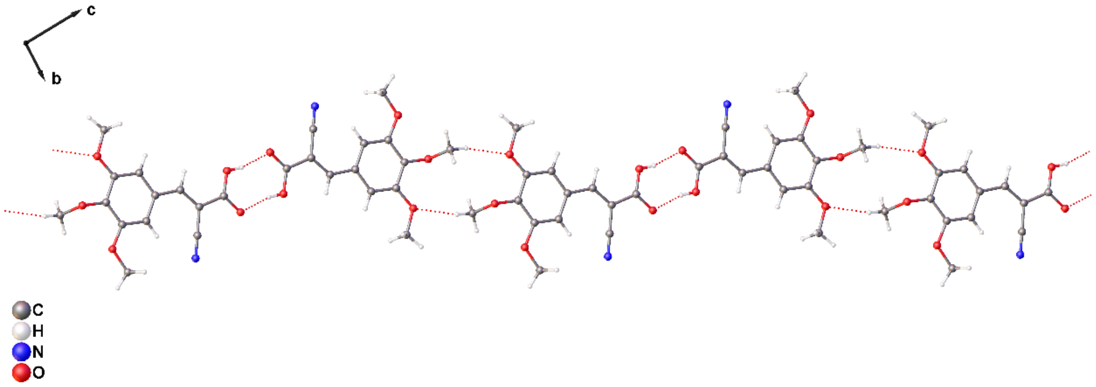

Figure S20. One-dimensional chain formed in **6** with the hydrogen bonds, viewed in the *bc* plane.
